# FXa‐Responsive Hydrogels to Craft Corneal Endothelial Lamellae

**DOI:** 10.1002/adhm.202402593

**Published:** 2025-01-22

**Authors:** Mikhail V. Tsurkan, Juliane Bessert, Rabea Selzer, Sarah D. Tsurkan, Dagmar Pette, Manfred F. Maitz, Petra B. Welzel, Carsten Werner

**Affiliations:** ^1^ Max Bergmann Center of Biomaterials Dresden Leibniz‐Institut für Polymerforschung Dresden e. V. Hohe Str. 6 01069 Dresden Germany; ^2^ TissueGUARD GmbH Trienter Str 16 01217 Dresden Germany; ^3^ Faculty of Medicine Carl Gustav Carus Institute of Anatomy Technische Universität Dresden Fetscherstr. 74 01307 Dresden Germany; ^4^ Else Kröner Fresenius Center for Digital Health University Hospital Carl Gustav Carus Dresden Technische Universität Dresden Fetscherstr. 74 01307 Dresden Germany; ^5^ Center for Regenerative Therapies Dresden Technische Universität Dresden Fetscherstr. 105 01307 Dresden Germany

**Keywords:** biorthogonal, corneal endothelial lamellae, hydrogel, stimuli‐responsive hydrogel, tissue engineering

## Abstract

Cell‐instructive polymer hydrogels are instrumental in tissue engineering for regenerative therapies. Implementing defined and selective responsiveness to external stimuli is a persisting challenge that critically restricts their functionality. Addressing this challenge, this study introduces a versatile, modular hydrogel system composed of four‐arm poly(ethylene glycol)(starPEG)‐peptide and glycosaminoglycan(GAG)‐maleimide conjugates. The gel system features a small peptide sequence that is selectively cleaved by the coagulation factor FXa. In a cell culture environment, where active FXa is absent, the hydrogel remains stable, providing a conducive matrix for the growth of complex tissue structures or organoids. Upon the introduction of FXa, the hydrogel is designed to disintegrate rapidly, enabling the gentle release of the cultivated tissues without impairing their functionality. The efficacy of this approach is demonstrated through the ex vivo development, detachment, and transplantation of human corneal endothelial lamellae, achieving sizes relevant for clinical application in Descemet Membrane Endothelial Keratoplasty (DMEK). Furthermore, the practicality of the hydrogel system is validated in vitro using a de‐endothelialized porcine cornea as a surrogate recipient. Since the FXa‐cleavable peptide can be integrated into a variety of multifunctional hydrogels, it can pave the way for next‐generation scaffold‐free tissue engineering and organoid regenerative therapies.

## Graphical abstract

This study introduces a sacrificial, multicomponent biohybrid hydrogel system enabling stable scaffold‐free tissue engineering. Its efficacy is demonstrated through the ex vivo development, detachment, and transplantation of human corneal endothelial lamellae, achieving clinically relevant sizes for Descemet Membrane Endothelial Keratoplasty. This versatile approach advances scaffold‐free tissue and organoid engineering, paving the way for translation into regenerative therapies.

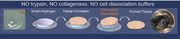

## 1 Introduction

Introduction. Emerging biotechnological innovations continually expand the scope of human tissue constructs that can be produced ex vivo, setting the stage for their application in regenerative therapies.^[^ ^1^, ^2^ ^]^ This development necessitates materials that can support tissue and implant production, with particular emphasis on multi‐component cell culture carriers that offer defined signaling characteristics to guide functional tissue formation. Strong cell adhesion to these materials is typically essential. However, after tissue formation, it should be gently detached from the supporting material while preserving its cellular and extracellular matrix (ECM) structure. Cell adhesion in cell‐instructive materials is facilitated by integrin‐binding ligands akin to the ECM of the formed tissue. Chemical or enzymatic dissociation of these bonds could cause substantial tissue damage. Consequently, innovative biorthogonal strategies are needed to selectively separate the formed tissue from the material without causing any damage. This technology could greatly benefit the creation of delicate tissues like corneal endothelial lamellae intended for corneal transplantation. Despite two decades of rigorous efforts, these tissues have not yet been successfully formed in sizes suitable for transplantation, and attempts at transplantation with a scaffold have been unsuccessful.

Common cell detachment and dissociation methods often employ ECM‐digesting enzymes such as trypsin, pepsin, and matrix metalloproteases (MMPs) like collagenases or non‐enzymatic ECM‐chelation buffers. However, these approaches are not suitable for the release of engineered tissues due to their destructive impact on the crucial ECM structure of these tissues. Moreover, these enzymes can cause unwanted cleavage of cell surface proteins, further compromising the integrity of the tissue.^[^ ^3^, ^4^ ^]^

Seeking an alternative to traditional enzymatic methods for cell‐material detachment, Okano and his team pioneered the use of temperature‐responsive hydrogels. These hydrogels, primarily made from poly(N‐isopropylacrylamide) (pNIPAM), introduce mechanical stress when subjected to temperature changes. Specifically, a drop in temperature from 37  to 10 °C causes the polymer coating to swell. This induced mechanical stress subsequently facilitates the detachment (or delamination) of adherent cells.^[^ ^5^, ^6^ ^]^ However, the potential of temperature‐responsive materials is somewhat curtailed by their limited compatibility with peptides, proteins, or carbohydrates, thereby restricting their ability to control cell fate. Additionally, temperature fluctuations could potentially impair the cells. The mechanical stress induced by these temperature changes often risks tearing the newly formed tissue.^[^ ^7^ ^]^ Consequently, while temperature‐responsive materials have proven to be a powerful research tool, their use in clinically applied technologies has remained limited.^[^ ^8^ ^]^

Recently, various non‐temperature‐responsive systems have been employed to gather cells and scaffold‐free cell monolayers while minimally affecting their ECM integrity. These systems include ion‐induced cell‐detachment^[^ ^9^ ^]^ and electro‐responsive,^[^ ^10^ ^]^ photo‐responsive,^[^ ^11^ ^]^ and pH‐responsive^[^ ^12^ ^]^ materials. Additionally, nanocellulose‐based biomaterials are gaining recognition as sacrificial scaffolds.^[^ ^13^ ^]^ Detailed reviews have been conducted on the advantages and disadvantages of these systems and their respective methodologies.^[^ ^14^, ^15^ ^]^ However, the common challenges of physical or chemical stress and limitations in (bio)molecular functionalization persist across these different approaches.

Synthetic hydrogel scaffolds, which offer precise control over multiple biochemical properties (like biodegradation, adhesion ligands, soluble effectors, etc.) independent of their physical attributes, hold significant promise for tissue engineering technologies.^[^ ^16^ ^]^ By utilizing pharmaceutical‐grade components such as synthetic polymers, peptides, and biomolecules, these materials can be transitioned into clinical applications. This presents a notable advantage over the frequently used nature‐derived products (such as basement membrane extracts), which are limited by their ill‐defined and variable composition. The incorporation of stimuli‐responsive features into chemically defined, cell‐instructive hydrogels, like on‐demand biorthogonal degradability, can offer unprecedented functionality, paving the way for both the creation and extraction of engineered tissues or organoids ex vivo.

In pursuit of this goal, we drew inspiration from recombinant protein production. In this process, high‐affinity tags are engineered into the protein structure–either at the C‐terminus or N‐terminus–to facilitate the cost‐effective purification of recombinant proteins from a complex blend of intracellular and extracellular biopolymers using affinity chromatography.^[^ ^17^, ^18^ ^]^ The protein tag is typically connected to the recombinant protein via a short enzymatically cleavable peptide sequence. This allows for the careful, selective removal of the tag by the respective enzyme–most commonly proteases without affecting the protein structure.^[^ ^19^ ^]^ These tag removal enzymes (Table S1, Supporting Information) cleave specific amino acid sequences with very high selectivity and specificity, rendering them inactive toward common cellular and ECM proteins.

Inspired by this concept, we developed novel sacrificial biomaterials with bioorthogonal responsiveness by integrating a small peptide motif, cleavable by tag removal enzymes, into the hydrogel network.^[^ ^20^ ^]^ Specifically, we designed multifunctional GAG‐based hydrogels featuring a peptide linker that can be cleaved by the blood coagulation factor FXa. Known as one of the most commonly employed tag enzymes, FXa protease exhibits a high cleavage activity while maintaining considerable selectivity.^[^ ^21^ ^]^ Leveraging this approach, we have successfully combined the benefits of a previously established modular cell‐instructive hydrogel platform with a biorthogonal, enzymatically cleavable peptide crosslinker. The expectation that this allows for efficient and selective degradation of the biomaterial without causing any harm to the attached cells or tissue is further supported by a recent report on the use of FXa‐responsive hydrogels for the in vitro culture and release of mesenchymal stroma cells.^[^ ^22^ ^]^

**Figure 1:**
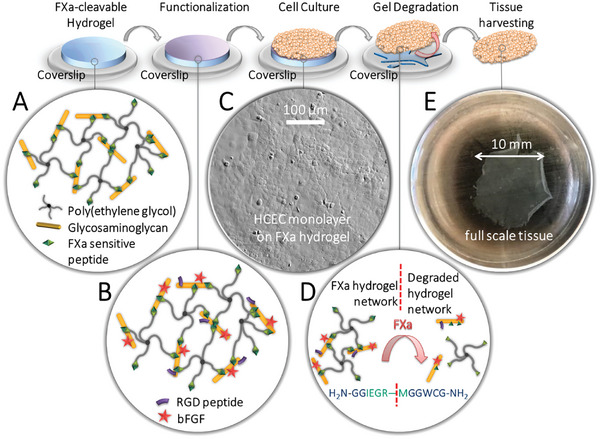
Schematic summary of the introduced biorthogonal approach for the culture and gentle harvest of cells and tissues using FXa‐degradable biohybrid hydrogels: A) The biohybrid hydrogel is composed of (starPEG)‐maleimide and the glycosaminoglycan chondroitin sulfate (CS)‐maleimide. The FXa‐cleavable peptide sequence H_2_N‐GGIEGR‐MGGWCH‐NH_2_ covalently crosslinks both components. Discs of these FXa‐degradable biohybrid hydrogels can be prepared on glass coverslips. B) The FXa‐degradable biohybrid hydrogels can be functionalized (via covalent or electrostatic interactions) with various biological moieties, e.g. the adhesion‐mediating peptide sequence RGD or basic fibroblast growth factor (bFGF), which support the initial cell adhesion or later tissue formation and growth. C) In the following step, cells can be seeded onto the hydrogel. D) After forming a functional tissue, the incubation of the samples with the enzyme FXa under physiological conditions leads to the specific cleavage of the FXa‐cleavable peptide sequence linking starPEG‐maleimide and CS‐maleimide. E) This procedure results in the complete decomposition of the sacrificial biohybrid hydrogel and in the gentle release of the tissue. Cell‐cell‐contacts, cell‐matrix‐contacts, and cell‐surface proteins are not impaired.

In our quest to further explore this technology's potential, we chose modular biohybrid hydrogel materials containing sulfated glycosaminoglycans (sGAGs) as our foundation. These materials have been demonstrated to recapitulate multiple regulatory functions of the extracellular matrix, particularly due to their proteoglycan‐like capacity to reversibly complex a broad range of crucial soluble signaling molecules, growth factors, and cytokines.^[^ ^23^, ^24^ ^]^ The respective starPEG‐sGAG platform has been successfully applied to induce the formation of capillary networks of vascular endothelial cells,^[^ ^25^ ^]^ of kidney proximity tubular tissue,^[^ ^26^ ^]^and epithelial tubular genesis.^[^ ^27^ ^]^ In this approach, starPEG‐peptide conjugates and sGAG‐peptide conjugates are tailored to assemble them via a Michael‐type addition reaction into covalently connected hydrogel networks of thoroughly programmed physical and biomolecular characteristics of ECMs.^[^ ^28^, ^29^ ^]^ In the herein‐reported extension of this material platform, we have replaced MMP‐cleavable peptide crosslinkers with FXa‐cleavable ones. The new crosslinkers remain stable during cell culture but are quickly cleaved in the presence of the enzyme FXa (**Figure** **1**; Figure S10, Supporting Information). FXa, a serine protease of the blood coagulation cascade, originates as an inactive zymogen (FX) in the liver. It, which necessitates interaction with either FVIIa or FIXa, only takes place during blood coagulation. As such, active FXa is not present in healthy tissue or cell cultures.^[^ ^30^, ^31^ ^]^ The introduction of FXa‐cleavable peptide conjugates into the hydrogel polymer network allows for the “on‐demand” gentle release of the tissue construct, while the high substrate specificity of this protease minimizes potential disruption to extracellular matrix organization, thereby enabling biorthogonal hydrogel degradation. Nevertheless, potential cellular responses triggered by the activation of protease‐activated receptors (PAR), specifically PAR‐1, PAR‐2, and PAR‐3, must also be considered.

**Figure 2:**
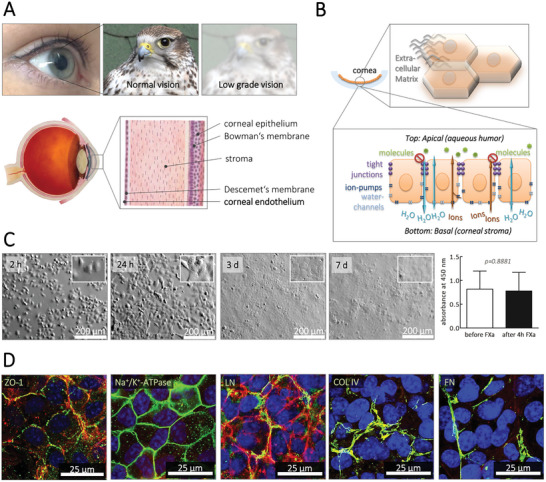
The human corneal endothelium and culture of human corneal endothelial cells (hCEnC) on FXa‐degradable hydrogels: A) The cornea anatomy. Five different layers can be distinguished – the anterior stratified squamous corneal epithelium, which resides on the Bowman's membrane; the corneal stroma; and the corneal endothelium, which resides on the Descemet's membrane. Injuries or diseases of the cornea, especially of the corneal endothelium, can lead to corneal edema, resulting in corneal blindness. B) The morphological and biofunctional characteristics of the corneal endothelium. C) hCEnC adhered to the RGD‐functionalized FXa‐degradable hydrogels and started spreading already 2 h after seeding. After seven days of culture at 37 °C in a humidified atmosphere containing 5% CO_2_ a confluent cell layer composed of small polygonal cells had been formed. The culture of hCEnC on FXa‐degradable hydrogels and treatment of the cells with 3600 nm FXa did not impair cell metabolism. D) hCEnC cultured for seven days on FXa‐degradable hydrogels were positive for tight junction Zonula occludens‐1 (ZO‐1) and for the ion‐pump Na^+^/K^+^‐ ATPase α1, which were both localized at the lateral cell membranes. Moreover, they expressed fine fibers of laminin, collagen type IV, and fibronectin as typical components of the extracellular matrix of the corneal endothelium. Antigens of interest are shown in green (Alexa Fluor^®^488), F‐actin fibers in red (Phalloidin), and the nuclei in blue (Hoechst 33342).

We demonstrate the potential of the new hydrogels with bio‐orthogonal release functionality by applying them to the bioengineering of corneal endothelial lamellae. Corneal endothelial cells (hCEnC) form a single‐cell tissue layer on the posterior cornea. Mature hCEnCs have limited to no regeneration potential, and their dysfunction is a primary cause of cornea transplantation (**Figure** **2**A).^[^ ^32^ ^]^ Globally, ≈12.7 million individuals suffer from corneal blindness,^[^ ^33^ ^]^ a condition that could be cured with cornea transplantation. The common treatment modalities are penetrating^[^ ^34^ ^]^ (full thickness) and lamellar^[^ ^35^ ^]^ (partial) corneal keratoplasty (transplantation). However, the scarcity of usable corneal donor tissue necessitates the development of alternative therapeutic approaches based on bioengineered tissues. To this end, several technologies are being explored. For instance, cornea constructs were bioprinted to match the specific shape and size of a patient's cornea.^[^ ^36^, ^37^, ^38^ ^]^ Unfortunately, 3D‐printed corneas cannot support hCEnC, which loses its morphological characteristics on the 3D‐printed materials. Current 3D‐printed cornea approaches focus on Deep Anterior Lamellar Keratoplasty (DALK), which replaces only the epithelium and stroma part of the cornea. Accordingly, 3D‐printed cornea constructs can be applied only to patients with intact Descemet membranes and functional endothelium cells, which account for less than 30% of cornea transplantation cases. This limitation excludes up to 70% of the patients suffering from cornea blindness.^[^ ^39^ ^]^

The feasibility of creating a lamellar corneal transplant is underscored by the fact that the lamella is a monolayer tissue. Descemet Membrane Endothelial Keratoplasty (DMEK) removes the patient's dysfunctional Descemet membrane and endothelium, substituting it with a corneal endothelial lamellae transplant from a donor cornea. DMEK surgery stands out as one of the most swift and minimally invasive methods to rectify endothelial dysfunction. Any lab‐created tissues intended for use in DMEK operations need to be at least 0.6 cm in diameter to adequately replace donor tissues. Furthermore, the generation of a functional DMEK implant necessitates the preservation of hCEnC morphological characteristics, such as its hexagonal shape and functional proteins, including tight junction protein Zonula occludens‐1 (ZO‐1) and ion‐pump Na+/K+‐ ATPase α1, both of which are localized at the lateral cell membranes Figure 2B.^[^ ^40^, ^41^ ^]^ The presence of specific ECM proteins of the Descemet membrane, including laminins, collagen IV, and fibronectin, is also necessary. Despite attempts over several decades to produce hCEnC ex vivo for transplantation, progress toward clinical translation has been limited. This is due to the need for not only specific microenvironmental cues to maintain the cell characteristics but also technologies that allow for the retrieval of the engineered tissue from its culture substrate.^[^ ^39^ ^]^

## 2 Results and Discussion

Our biomaterial‐based technology offers a dual advantage: it provides a multi‐biofunctional cell‐instructive environment to nurture and guide cells, and leverages a bio‐orthogonal degradation approach for the retrieval of the formed tissue. We derived corneal lamellar implants suitable for DMEK from the HCEC‐B4G12^[^ ^42^ ^]^ human corneal endothelium cell line, which shows morphological characteristics similar to primary cells. The FXa cleavable starPEG‐chondroitin sulfate hydrogel, functionalized with the cell adhesive cyclic RGD peptide, served as the culture support, recapitulating the composition of chondroitin‐containing stroma and Descemet membrane which consists of laminins and fibronectin (Figure 1A,B). In addition, the hydrogel was loaded with bFGF via the sGAG's electrostatic interaction to promote hCEnC expansion. Adherence and spreading of the cells were observed already two hours after seeding. Even under serum‐free conditions, the hCEnC cell line formed a confluent monolayer with a uniform hexagonal morphology after seven days of culture (Figure 2C). Immunostaining revealed the presence of characteristic tight junction (ZO‐1) and ion‐pump (Na^+^/K^+^‐ ATPase α1) proteins at the lateral cell borders, resembling natural tissue (Figure 2D; Figures S16–S18, Supporting Information). Moreover, the formed cell sheets expressed fine fibers of laminin, collagen type IV, and fibronectin, which are the main components of the extracellular matrix of corneal endothelium (Figure 2D). Collectively, the morphological and immunostaining characteristics of the hCEnC cell sheets engineered on the surface of FXa‐degradable starPEG‐CS hydrogels were found to closely resemble those of native corneal lamella. Thus, our proposed method allows cells to maintain their cell‐cell junction, cell surface proteins, and extracellular matrix.

In assessing the safety of our novel biomaterial‐based technology, we have taken into account that FXa, beyond its role in blood coagulation, is also implicated in various inflammatory processes, vascular remodeling, and tissue fibrosis.^[^ ^43^ ^]^ Notably, FXa activates PAR‐1, PAR‐2, and PAR‐3. PAR‐1 activation by thrombin and FXa results in phosphorylation of the myosin light chain II and a consequent loss of barrier integrity of the cell layers.^[^ ^43^, ^44^ ^]^ However, this effect, which does not impact cell viability, dissipates quickly as FXa is deactivated by antithrombin III from body fluids such as blood or human aqueous humor within minutes in vivo. To examine the impact of FXa exposure on hCEnC in culture, we exposed hCEnC, grown on cell laminin coatings, to FXa. We observed no effect on the metabolic activity, even when applying FXa concentrations four times higher (3.6 µm) than those used for the hydrogel cleavage (Figure 2C). These findings underscore the safety of using FXa for the collection of hCEnC lamellae.

**Figure 3:**
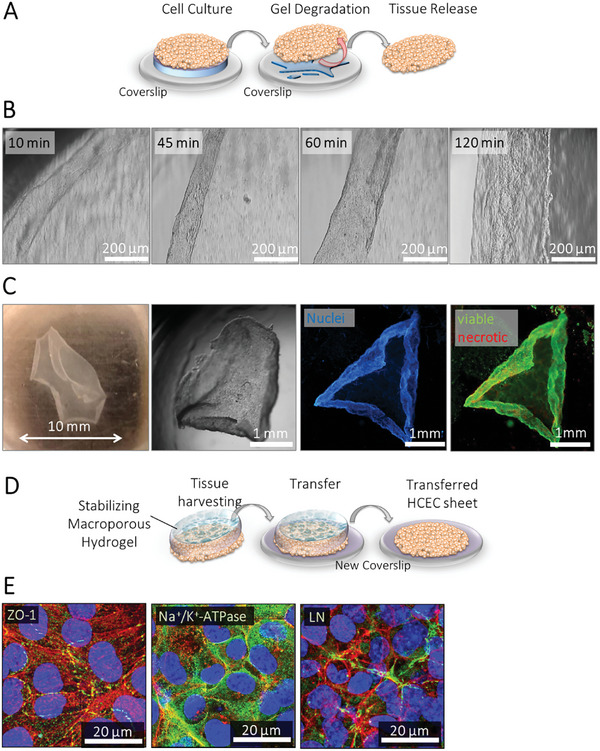
Harvest and transfer of cultured hCEnC sheets from FXa‐degradable biohybrid hydrogels onto a new planar substrate using macroporous biohybrid hydrogels. A,B) After seven days of culture under serum‐free conditions, the hCEnC cell line formed a confluent monolayer. Upon incubation with 900 nm FXa enzyme in a serum‐free medium for at least 90 min at 37 °C, the cellular monolayer was completely released from the (degraded) biohybrid hydrogel. C) This release process of the hCEnC monolayer is accompanied by a shrinking of the tissue in its size to ≈60%. Depending on the original size of the FXa‐degradable biohybrid hydrogel, various cell sheet sizes can be generated. Life‐dead‐staining of the released cell layer showed that the majority of the cells were still viable after the detachment. Viable cells are shown in green (Calcein‐AM), and necrotic cells in red (PI). D) Released cellular monolayers were stabilized by discs of macroporous biohybrid hydrogels. This allowed for the manipulation of the fragile tissue and its transfer onto a new planar target surface. E) One day after the transfer onto the new planar target surface, the hCEnC monolayers were positive for the function‐associated marker proteins ZO‐1 and Na^+^/K^+^‐ATPase (shown in green), which were detected at the lateral cell borders, and for the ECM protein laminin (shown in green). F‐actin fibers are shown in red (Phalloidin), and the nuclei in blue (Hoechst 33342).

Validating the functionality of our system, we demonstrated that only 900 nM FXa, added to a serum‐free medium for 90 min at 37 °C, was sufficient to harvest the intact cellular monolayer from the hydrogel scaffold (**Figure** **3**A,B). The time required for the cleavage of the hydrogel allowed for monitoring the detachment and upscrolling of hCEnC cell sheets, similar to the scrolling of the native DMEK graft corneal lamella from the cornea before implantation (Figure 3B; Figures S13 and S14, Supporting Information). The released hCEnC monolayers shrank to ≈60% of the original diameter (9‐6 mm). This shrinkage, attributed to the forceful pulling actions of the cells via tight junction proteins, confirms that these junctions were maintained during FXa treatment, as also evidenced by immune staining (Figure 3E). Remarkably, the harvested tissue construct exhibited a rolling behavior akin to Pre‐Descemet's endothelial keratoplasty (PDEK), which may be due to stronger tight junction protein interactions and an immature Descemet membrane in the newly formed tissue. Live‐dead‐staining of the released cell layer indicated that the majority of the cells were viable after the monolayer detachment from the hydrogel substrate (Figure 3C; Figure S19, Supporting Information). We used a disc of a soft, yet robust macroporous biohybrid hydrogel (cryogel)^[^ ^45^ ^]^ to stabilize the formed fragile tissue as a flat construct and to transfer it onto a new planar target surface (Figures S20 and S21, Supporting Information). Importantly, the main morphological characteristic, functional proteins like ZO‐1 and the Na^+^/K^+^ ATPase, as well as the ECM of the tissue, remained unaltered (Figure 3D,E). The orientation of F‐actin fibers shows that neither the FXa treatment nor the transfer process impaired cell‐cell and cell‐matrix contacts.

**Figure 4:**
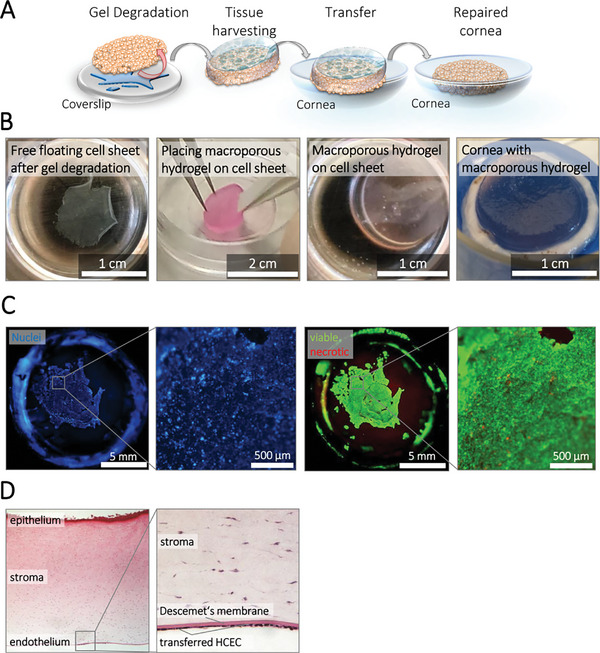
Harvest and transfer of cultured hCEnC sheets from FXa‐degradable biohybrid hydrogels onto a new concave de‐endothelialized porcine cornea using macroporous biohybrid hydrogels. A,B) After seven days of culture under serum‐free conditions, the hCEnC monolayer was released from the biohybrid hydrogel by incubating the samples with 900 nm FXa enzyme in a serum‐free medium for at least 90 min at 37 °C. Released cellular monolayers were stabilized by discs of macroporous biohybrid hydrogels and transferred onto the new concave target surface–a de‐endothelialized porcine cornea. The macroporous biohybrid hydrogel (cryogel) nicely adapted to the curvature of the concave cornea. C) Staining of the cell nuclei and life‐dead staining nicely showed the successful transfer of the hCEnC sheet. The majority of the cells were viable one day after the transfer. Viable cells are shown in green (Calcein‐AM) and necrotic cells in red (PI). D) The cross‐section of the cornea shows the cells that appeared to be attached to Descemet's membrane as a confluent monolayer.

To illustrate the potential clinical applicability of our proposed approach, we conducted an in vitro model transplantation experiment utilizing a freshly prepared de‐endothelialized porcine cornea as a model recipient (**Figure** **4**A,B). We were able to manipulate and fix released hCEnC sheets of ≈1 mm in diameter into the required shape using macroporous biohybrid hydrogels.^[^ ^45^ ^]^ These hydrogels adapted well to the curvature of the concave cornea and facilitated a successful cell sheet transfer, as visualized by staining of the cell nuclei (Figure 4C; Figures S24 and S25, Supporting Information). Live‐dead staining revealed that the majority of the cells remained viable after one day of culture (Figure 4C). Furthermore, a cross‐section of the cornea showed the hCEnC sheets attached to the Descemet membrane as a dense monolayer, indicating successful transplantation (Figure 4D; Figure S26, Supporting Information). This suggests that our approach could be a promising avenue for future clinical applications in corneal transplantation.

To demonstrate the versatility of the biomaterial‐based technology, we also employed cultures of primary human umbilical vein endothelial cells (HUVECs) and primary mesenchymal stem cells (MSC). Similar to the previous experiments, we were able to form monolayer cell sheets on the hydrogel materials and retrieve them intact and viable in centimeter dimensions by administering FXa. These retrieved cell sheets were stabilized by a macroporous hydrogel disc, as described above (Figure S27, Supporting Information). In essence, these findings confirm the efficiency of the proposed technology for ex vivo tissue transplant fabrication, and the stabilization and transfer of delicate constructs to target surfaces of various shapes. Although we utilized our previously established starPEG‐sGAG hydrogel platform in this synthetic design, the principle can be readily applied to other peptide‐containing polymer networks, as demonstrated in previous studies.

## 3 Conclusion

In summary, we have successfully adapted and validated our bio‐orthogonal technology for the use of multi‐biofunctional hydrogel materials in the ex vivo production of transplantable tissues without any artificial scaffold. By combining cell‐instructive biohybrid hydrogels with a protein TAG‐removal concept, we facilitated the formation and harvesting of corneal lamella tissue suitable for surgical implantation in Descemet membrane endothelial keratoplasty (DMEK). The collection and transfer of the delicate corneal lamella tissue onto both flat and curved surfaces were made possible through the use of a macroporous biohybrid hydrogel. This approach has the potential to replace donor tissue in DMEK surgeries.

Importantly, our method allows cells within the formed tissue graft to maintain their cell‐cell junctions, cell surface proteins, and extracellular matrix as they exist in the native tissue. While our material design is optimized for corneal lamella graft production, our technology can be expanded to functionalize modular hydrogel materials,^[^ ^46^ ^]^ embedding cells to form tissue‐like or organoid cultures. The disclosed biotechnology facilitates the efficient, scaffold‐free harvesting of fully functional dimensional tissue and organoid constructs, which is not achievable by other means.

## 5 Conflict of Interest

The authors declare no conflict of interest.

## 6 Author Contributions

M.V.T. initiated and designed the study, performed the hydrogel synthesis and the related analysis, supervised R.S., and wrote the manuscript. J.B. designed and performed the cornea transfer experiments, supervised R.S. and D.P., and wrote the manuscript. RS performed the hydrogel synthesis, related analysis, and cell experiments and analyzed the data. D.P. and S.D.T. helped with cell and cornea transfer experiments. PBW designed and synthesized the cyrogel tool, discussed data, and edited the manuscript. M.F.M. and C.W. discussed data and edited the manuscript.

7

## Supporting information



Supporting Information

## Data Availability

The data that support the findings of this study are available in the supplementary material of this article.
