# Supplementary material for: FXa‐Responsive Hydrogels to Craft Corneal Endothelial Lamellae
Source: Adv Healthc Mater. 2025 Jan 22;14(10):2402593. doi: 10.1002/adhm.202402593 (PMC12004427; doi:10.1002/adhm.202402593)
Supplement: Supplementary file 1 — Supporting Information [file ADHM-14-0-s001.docx]

Supplementary Materials for

**FXa-Responsive Hydrogels to Craft Corneal Endothelial Lamellae**

Mikhail V. Tsurkan^1,4#^*, Juliane Bessert^#1,3^, Rabea Selzer^#1^, Sarah D. Tsurkan^4,5^, Dagmar Pette^1^, Manfred F. Maitz^1^, Petra B. Welzel^1^, Carsten Werner^1,2^

^#^equal contribution *Correspondence to: [tusrkan@ipfdd.de](mailto:tusrkan@ipfdd.de)

^1^ Leibniz-Institut für Polymerforschung Dresden e.V., Max Bergmann Center of Biomaterials Dresden, Hohe Str. 6, 01069 Dresden, Germany

^2^ Technische Universität Dresden, Center for Regenerative Therapies Dresden, Fetscherstr. 105, 01307 Dresden, Germany

^3^ Technische Universität Dresden, Faculty of Medicine Carl Gustav Carus, Institute of Anatomy, Fetscherstr. 74, 01307 Dresden, Germany

^4^ TissueGUARD GmbH, Trienter Str 16, 01217 Dresden, Germany

^5^ Else Kröner Fresenius Center for Digital Health, University Hospital Carl Gustav Carus Dresden, Technische Universität Dresden, Fetscherstr. 74, 01307 Dresden, Germany

INDEX

Materials and Methods S2

Reagents and Supplies S2

Peptide synthesis S2

Preparative high performance liquid chromatography (prepHPLC) S3

Analytical high performance liquid chromatography (HPLC) S3

Analytical high performance liquid chromatography (HPSEC) S3

Electrospray ionization mass spectrometry (ESI-MS) S3

Synthesis of PEG-peptide conjugate PEG-(FXa)_4_ S4

Synthesis of chondrotinsulfate-maleimide conjugate CSMal_6_ S4

Characterization of maleimide reactivity within CSMal_6_ conjugate S4

Hydrogel formation S5

Hydrogel setup for cell culture S5

Rheological measurements S5

Enzymatic degradation analysis S6

Preparation of macroporous hydrogel (cryogel) as transfer tools S6

Human corneal endothelial cell culture on FXa-degradable hydrogels S6

Formation of hCEnC layers through the hydrogel degradation S7

Transplantation of the formed hCEnC layers S7

Cell viability assays S7

Analysis of metabolic activity rates (WTS-1) S7

Immunocytochemistry S8

Histochemistry and immunohistochemistry S8

Data analysis and graphing S9

Supplementary figures S10

Supplementary Tables S28

References and Notes S29

Materials and Methods:

**Reagents and Supplies**

All solvents and reagents for peptide synthesis, purification and conjugation were purchased from IRIS Biotech GmbH (Marktredwitz, Germany). 1-ethyl-3-(3-dimethyl aminopropyl)carbodiimide (EDC), N-hydroxysulfo-succinimide (sNHS), laminin (LN), chondroitin-6-sulfate (CS), recombinant human basic fibroblast growth factor (bFGF), antibiotic antimycotic solution (10,000 units penicillin, 10 mg streptomycin, and 25 μg amphotericin B per ml), trypsin/ethylenediaminetetraacetic acid (EDTA), ProClin^®^300 Preservative for Diagnostic Reagents, paraformaldehyde, Triton X-100, Sigmacote®, poly(ethylene-alt-maleic anhydride) (PEMA), hematoxylin and eosin were purchased from Sigma-Aldrich (Munich, Germany). Complete™ Protease Inhibitor Cocktail and the cell proliferation reagent WST-1 were g/mol purchased from Roche Diagnostics/Sigma Aldrich (Munich, Germany). Heparin (Mw = 14,000 g/mol) was purchased from Merck (Darmstadt, Germany). Four-armed amine-terminated polyethylene glycol (starPEG; Mn = 10.0x10^3^; PDI = 1.08) was purchased from JenKem Technology USA Inc. (Allen, USA). All reagents were used without purification. Phosphate-buffered saline without Mg^2+^/Ca^2+^ ( PBS w/o Mg^2+^/Ca^2+^) and phosphate-buffered saline with 100 mg/L MgCl_2_ and 100 mg/L CaCl_2_ (PBS w/ Mg^2+^/Ca^2+^) were purchased from Biochrom AG (Berlin, Germany). The serum-free medium Human Endothelial-SFM, Alexa Fluor®488 goat anti-mouse IgG, Alexa Fluor^®^488 goat anti-rabbit IgG, Alexa Fluor®488 donkey anti-goat IgG, HOECHST 33342, and propidium iodide (PI) were purchased from Thermo Fisher Scientific (Darmstadt, Germany). Rabbit anti-human fibronectin IgG was purchased from Biomol, Rockland Immuno-Chemicals (Hamburg, Germany). Goat anti-human collagen type IV IgG was purchased from Biozol, Southern Biotech (Eching, Germany). Rabbit anti-human laminin IgG was purchased from Sigma-Aldrich (Munich, Germany). Mouse anti-human ZO-1 IgG (clone 1/ZO-1) was purchased from Becton Dickinson (Heidelberg, Germany). Mouse anti-human Na^+^/K^+^-ATPase α1 IgG (clone 464.6) was purchased from Abcam (Cambridge, UK). AttoFluor^®^633 phalloidin was purchased from ATTO-TEC (Siegen, Germany). Calcein acetoxymethyl ester (Calcein-AM) was purchased from PromoKine (Heidelberg, Germany). Factor Xa Endoprotease (Ile-Glu-Gly-Arg) from bovine plasma was purchased from New England BioLabs (Frankfurt am Main, Germany). Dextran T500 was purchased from Carl Roth (Karlsruhe, Germany). Porcine eyes for the preparation of de-endothelialized corneas were from the local slaughter Saubachtaler Agrar AG Fleischland (Klipphausen-Sora, Germany). Goat and donkey serum were purchased from Dianova GmbH (Hamburg, Germany). Anti-fading mounting medium was purchased from O. Kindler GmbH (Freiburg, Germany). DePex was purchased from Serva Electrophoresis GmbH (Heidelberg, Germany).

**Peptide Synthesis**

All peptides were synthesized by solid-phase methods and standard Fmoc-chemistry using an Activo P14 (Activotec, Cambridge UK) peptide synthesizer. Specifically, the 1mmol scale protocol with a C-terminal capping protection strategy by amide was used. Amino acid activation was achieved by O-(benzotriazol-1-yl)-N,N,N',N'-tetramethyluronium tetrafluoroborate (TBTU), and 1-hydroxybenzotriazole (HOBt) in DMF with diisopropylethylamine (DIPEA) as a base. Deprotection of the amino acid side chains and cleavage from the resin was performed by reaction with a mixture of trifluoroacetic acid (TFA) (87.5% v/v), phenol (5% v/v), tri-isopropyl silane (TIPS) (2.5% v/v), and water (5% v/v) for 3 hours at room temperature. The crude peptides were then precipitated in anhydrous diethyl ether, collected by vacuum filtration, and dried under vacuum. Final purification was achieved by preparative reversed-phase high performance liquid chromatography (HPLC). The sequences of all synthesized peptides are given **in Supplemental Figures S1 and S2.**

**Preparative high performance liquid chromatography (HPLC)**

All peptide purification was performed by HPLC on a reversed-phase Luna Preparative C-18 column (10 μM particle size, 30×250 mm; Phenomenex, U.S.A.). A linear gradient of water/acetonitrile containing 0.1 % (v/v) trifluoroacetic acid was used as the mobile phase. The HPLC separation runs were performed over 30 min using the flow rate of 20 ml/min and the monitoring wavelengths were set to a wavelength range of 210-278 nm. A two-pump system (1200 Series Agilent Technologies, Santa Clara, U.S.A.) equipped with an U.V./Vis diode array detector/spectrophotometer and a 1 cm path length cell was used. The collected peptides were lyophilized, and their purity was verified by analytical HPLC and electrospray ionization mass spectrometry. The analytical HPLC chromatograms and ESI-MS spectra of all synthesized peptides are given in **Supplemental Figures S1 and S2**.

**Analytical high performance liquid chromatography (HPLC)**

The analytical HPLC was performed on Phenomenex Luna 5u C-18 column (5μM particle size, 250×3 mm; Phenomenex, Torrance, U.S.A.). A linear gradient of water/acetonitrile containing 0.1 % (v/v) trifluoroacetic acid was used as the mobile phase. The HPLC analyses were performed over 40 min using the flow rate of 0.5 mL/min, and the monitoring wavelengths were set to a wavelength range of 210-278 nm. A two-pump system (1100 Series Agilent Technologies, Santa Clara, U.S.A.) equipped with a U.V./Vis detector/spectrophotometer with a 1 cm path length cell.

**Electrospray ionization mass spectrometry (ESI-MS)**

ESI-MS measurements were performed on Agilent Technologies 6230 TOF LC/MS spectrometer (Agilent Technologies, Santa Clara, U.S.A.) in line with a detector in the analytical HPLC instrument. Nitrogen was used as nebulizing and desolation gas.

**Analytical high performance size exclusion chromatography (HPSEC):**

HPSEC experiments were performed on BioSep-SEC-S 2000 column (Phenomenex, Torrance, U.S.A.). The peptide samples were eluted using standard phosphate-buffered saline (pH 7.4), with 0.5 mL/min flow rate, and monitored at 210-278 nm wavelength. To keep reproducibility of the results, purification of the columns using 10% DMSO was performed after each 20-30 runs. A two-pump system (1100 Series Agilent Technologies, Santa Clara, U.S.A.) equipped with a UV/Vis detector/spectrophotometer with a 1-cm path length cell was used.

**Synthesis of PEG-peptide conjugate PEG-(FXa)_4_**

Synthesis of PEG-(FXa)_4_ conjugate was performed as reported before [1]. Briefly, 427 mg of PEG-Mal was dissolved in 10 mL of 50 % (v/v) acetonitrile/water and mixed with 300 mg of the FXa peptide (30 % excess), which was dissolved in 15 mL of 50 % (v/v) acetonitrile/water. The pH of the reaction mixture was adjusted by 1 M NaOH to pH 7.5-8. The reaction was run for 5 hours under N_2_ atmosphere, and the completion of the reaction was followed by analytical HPLC. The reaction mixture was purified by preparative HPLC. The product was collected from the HPLC and freeze-dried for at least 24 hours. The formed white powder was then stored at -20 °C. The synthetic scheme and HPLC monitoring of the reaction are given **in Supplemental Figures S3 and S4**.

**Synthesis of chondroitin sulfate-maleimide conjugate CSMal_6_ (chondroitin sulfate containing six maleimide groups given as an example)**

110.6 mg of sNHS (at three-fold excess to the maleimide amine) and 243.3 mg of EDC (at five-fold excess) were added to a solution of 0.45 g of chondroitin sulfate (3.57x10^-3^ mmol) dissolved in ultrapure H_2_O. The reaction mixture was stirred at 5°C for 30 min. At this point, 64.5 mg of N-(2-aminoethyl)maleimide trifluoroacetate salt (2.15 x10^-2^ mmol) dissolved in 1 ml ddH_2_O (at 5 °C) was added, with the total volume of the reaction mixture 25 ml. The reaction was run overnight at room temperature. The CSM6 product was purified by dialysis (membrane with 5000-8000 molecular weight cut-off) against 1000 ml of 1M sodium chloride three times for at least 1 hour in order to remove any unreacted maleimide amine. This was followed by dialysis against 1000 ml of water at least four times for at least 1 hour. The product was then transferred into a round bottom flask and freeze-dried for at least 24 hours. The purified chondroitin sulfate-maleimide conjugate was stored at -20 °C. The purity of the formed conjugate was analyzed by HPSEC. The integration of the peak intensity at 278 nm (specific UV-absorbance of maleimide extinction coefficient about 490 mol^-1^cm^-1^ in aqueous solutions [2]) allows for the determination of bonded and unreacted "free" maleimide in chondroitin sulfate-maleimide conjugate samples. The synthetic scheme and HPSEC monitoring of the reaction are given in **Supplemental Figures S5 and S6**.

### Characterization of maleimide reactivity within chondrotin sulfate -maleimide CSMal_6_ conjugate

The reactivity of maleimide groups in the chondroitin sulfate conjugate was characterized by HPSEC. In this experiment, the chondroitin sulfate conjugate 4 mg/ml solution was mixed in PBS with the equal molar amount (to maleimide groups of CSMal_6_) of the RGDS peptide (molecular weight = 990 g/mol). The final concentration was adjusted to 4 mg/ml. The reaction was kept for at least 60 min at room temperature, and then 50 µl were injected in HPSEC. The elution time of the formed chondroitin sulfate-peptide conjugate was about 10-18 min, and the elution time of unreacted peptide 28 min. The integration of the peak intensity at 278 nm (specific UV-absorbance of tryptophan, extinction coefficient about 5500 mol^-1^cm^-1^. in aqueous solutions) allows for the determination of bonded and unreacted "free" peptide in the formed chondroitin sulfate-conjugate conjugate samples. It was found that less than 5 % of the initial peptide is present in the reaction mixture, which indicates >95% efficiency of the CSMal_6_ formation. An example of the quantification of maleimide groups in a chondroitin sulfate-maleimide conjugate with six maleimide groups is shown in **Supplemental Figures S7**.

### Hydrogel formation

The total solid content of all gels mixture was always kept at 5% (50 mg/ml) independent of the crosslinking degree (which is the molar ratio PEG/ CSMal_6_ = γ). Adjusting the volume ratio of the PEG-(FXa)_4_ and CSMal_6_, while keeping total volume constant allows the adjustment of the swelling and stiffness of the formed hydrogels as described before [1]. The schematic view of hydrogel formation and the mechanical properties as a function of hydrogels crosslinking degree are shown in **Supplemental Figures S8 and S9**.

### Hydrogel setup for cell culture

All hydrogel formation for cell experiments were formed with crosslinking degree γ=1.25 under sterile conditions beneath a sterile working bench. Sterile PBS w/o Mg^2+^/Ca^2+^ with 1% (v/v) antibiotic/antimycotic was used as the solvent. The hydrogels were prepared as 20 mm in diameter and 0.5 mm thick discs covalently attached to the coverslips as in general described before [3]. In detail, 2.79 mg CS-Mal_6_ were dissolved in 75 microliters of the solvent and mixed with 4,72 mg of PEG-(FXa)_4_ dissolved in 75 µL of solvent with the final pH = 5 (adjusted by 5 M HCl in order to prolong the gelation time). 150 µL of hydrogel reacting mixture was mixed by pipetting several times, immediately transferred on freshly prepared coverslips (d = 20 mm) coated with maleimide groups (for covalent hydrogel attachment, for details see reference [3]), and covered with coverslips (d = 18 mm) dip-coated with Sigmacote®. For the detailed description of the coverslip preparation, see [3, 4]. The gel formation occurred within several seconds, but let stay for 10 minutes in order to complete the reaction. Next, the top Sigmacote^®^-coated coverslip was carefully removed using flat forceps, and the bottom coverslip with the attached hydrogel was incubated for 4 h at 37°C with sterile PBS for 4 h ("empty" hydrogel) or PBS containing RGD peptide (RGD functionalized hydrogels). In order to get rid of antibiotic/antimycotic, the hydrogel was washed with sterile PBS w/o Mg^2+^/Ca^2+^ for several days. One day before cell seeding, sterile hydrogels were incubated in Human Endothelial-SFM w/ 100 ng/ml bFGF

### Rheological measurements

Oscillating measurements on swollen gel discs (PBS) were carried out on a rotational rheometer (ARES LN2; TA Instruments, Eschborn, Germany), fitted with a parallel plate geometry (plate diameter = 25 mm) as described before [1]. Frequency sweeps were performed at 25 °C with a shear frequency range of 10^-1^ – 10^2^ rad s^-1^ with a strain amplitude of 2%. Mean values of the storage modulus were calculated. Experiments were performed in triplicate. The gel volumetric swelling of the swollen hydrogel discs was determined by the following equation: Q=(D_f_/D_i_)^3^, where D_i_ is the initial diameter of the unswollen gel disc and D_f_ is the final diameter of the gel disc swollen for 24 hours in PBS. Hydrogel swelling and stiffness as the function of the crosslinking degree are shown in **Supplemental Figure S9**.

### Enzymatic degradation analysis

5 μl gel droplets were used for degradation experiments. After gelation, the hydrogels were washed three times and swollen in PBS overnight. To determine the kinetics of degradation, gel droplets were placed in plastic UV cuvettes (PlastiBrand, Wertheim, Germany) with either 1 ml of PBS or 900 nM FXa solution in PBS (MoBiTec GmbH, Germany or New England BioLabs, UK) at 37 °C. The UV absorption was measured at 278 nm using a UV/Vis spectrophotometer (Beckman Coulter DU800; Danvers, U.S.A.) and recorded for all samples every 2 min over a time period of 3 hours. Hydrogels degradation properties as the function of hydrogels crosslinking degree are shown in the **Supplemental Figure S10**.

### Preparation of macroporous hydrogel (cryogel) as transfer tools

Macroporous biohybrid hydrogels composed of starPEG and heparin with a cross-inking degree of γ = 2 were prepared according to the protocol described by Welzel *et al*. [5]. Dry macroporous biohybrid hydrogels with a diameter of 6 mm and a height of 1 mm were sterilized by incubation in sterile PBS w/o Mg^2+^/Ca^2+^ w/ 1 % (v/v) ProClin^®^300 preservative overnight. One day before the cell transfer experiment, the sterile macroporous hydrogel scaffolds were incubated in Human Endothelial-SFM w/ 10 ng/ml bFGF overnight at 37 °C. The schematic view of macroporous hydrogel (cryogel) disc formation for cell tissue transfer is shown in **Supplemental Figure S20 and S21**.

### Human corneal endothelial cell culture on FXa-degradable hydrogels

The immortalized human corneal endothelial cell (hCEnC) line HCEC-B4G12 [6] was cultured in Human Endothelial-SFM supplemented with 10 ng/ml human recombinant bFGF. Cells were subcultured by trypsinization using 0.05% (v/v) trypsin/0.02% (v/v) EDTA and seeded at a density of 10×10^4^ cells per cm^2^ on T75 culture flasks coated with 60 μg laminin and 3 mg chondroitin-6-sulfate. Cells were collected in Human Endothelial-SFM containing proteinase inhibitor cocktail (1 tablet in 50 ml medium) and centrifuged for 5 min at 700 rpm. hCEnC were seeded at a density of 1×10^5^ cells per cm^2^ onto the FXa-degradable hydrogels. The cells were maintained at 37 °C in a humidified atmosphere containing 5 % CO_2_. The medium was changed three times per week. Adhesion, proliferation and monolayer formation were documented by repeated imaging over seven days using Hoffman modulation contrast microscopy (Olympus IX**73** by Olympus GmbH, Hamburg, Germany) with a DP26 digital camera driven by CellSens Standard 1.8 software (Olympus GmbH, Hamburg, Germany). The schematic view of hydrogel formation and the mechanical properties as a function of hydrogel crosslinking degree are shown in **Supplemental Figures S11 and S12**.

**Formation of hCEnC layers through the hydrogel degradation**

After seven days of culture on the FXa-degradable hydrogels, the samples were incubated in Human Endothelial-SFM w/ 900 nM Factor Xa Endoprotease for 45 min at 37 °C. After this time, the cellular monolayers were completely released and could be carefully manipulated by using a 20 gauge cannula (HSW Fine Ject, Tuttlingen, Germany). The images of the HCEnC layers formation via the hydrogel degradation are shown in **Supplemental Figures S13 and S14**.

### Transplantation of the formed hCEnC layers

Macroporous biohybrid hydrogels were placed onto the released HCEnC layers. In that way, the hCEnC layers were stabilized and could be transferred either onto fibronectin-coated PEMA-coverslips (for detailed preparation, see Pompe *et al*.[4]) or onto de-endothelialized porcine corneas (for detailed preparation see Teichmann *et al*. [7]). The corneas with the transferred hCEnC layers were cultured in Human Endothelial-SFM w/ 10 ng/ml human recombinant bFGF and w/ 6% (w/v) dextran T500 at 37°C in a humidified atmosphere containing 5 % CO_2_. The medium was changed three times per week. The hCEnC layers transplanted onto de-endothelialized porcine corneas are shown in **Supplemental Figures S22 and S23**.

### Cell viability assay (life/dead)

In order to discriminate between viable and necrotic cells, hCEnC layers before and after the enzymatically induced release and transfer were incubated in a staining solution of 3.2 µM Calcein-AM (viable cells), 2.5 µM PI (necrotic cells), and 2 µg ml^-1^ HOECHST 33342 (cell nuclei) in sterile PBS w/ Mg^2+^/Ca^2+^ for 30 min at 37°C in a humidified atmosphere containing 5 % CO_2_. Immediately after staining, samples were photo-documented under a fluorescence microscope Axio Observer.Z1 with Zen lite image processing software (Jena, Germany). The life/dead staining of HCEnC layers before after hydrogel degradation are shown in the **Supplemental Figures S15 and S19.** The life/dead staining of de-endothelialized porcine corneas before and after the hCEnC layers transplantation are shown in **Supplemental Figures S24 and S25**.

**Analysis of metabolic activity rates (WTS-1)**

Metabolic activity rates of hCEnC after seven days of growth on the FXa-degradable hydrogels and exposure to FXa Endoprotease (New England BioLabs, UK) 900 nM concentration for 24 hours were examined using the cell proliferation reagent WST -1 according to the manufacturer's instructions. Supernatants were collected after 30 min incubation with WST-1 **(water-soluble tetrazolium salts)** supplemented Human Endothelial-SFM at 37°C in a humidified atmosphere containing 5 % CO_2_. Absorbance was determined at 450 nm (Tecan GENios Microplate Reader and universal data processing software Magellan 6 by Tecan Group Ltd., Männedorf, Switzerland). Three replicates were performed, and data were expressed as mean ± standard deviation.

**Immunocytochemistry**

Protein production in hCEnC grown for seven days on FXa-degradable hydrogels was analyzed by immunocytochemical staining before and 24 hours after transfer onto the PEMA-coated coverslips. For that, samples were rinsed with sterile PBS w/ Mg^2+^/Ca^2+^, fixed in 4 % paraformaldehyde at 37 °C for 15 min, and permeabilized with 0.5 % Triton X-100 at room temperature (RT) for 10 min followed by nuclear staining with 2 µg ml^-1^ HOECHST 33342 for 10 min. The samples were blocked in 10 % (v/v) goat or donkey serum in PBS w/ Mg^2+^/Ca^2+^ for 30 min, followed by incubation with the primary antibodies diluted in either 1 % (v/v) goat or donkey serum at R.T. hCEnC were stained for the ECM constituents fibronectin (dilution 1:200), collagen type IV (dilution 1:20) and laminin (dilution 1:20), the tight junction protein ZO-1 (dilution 1:100) and the ion-pump Na^+^/K^+^-ATPase α1 (dilution 1:10). The samples were again blocked for 30 min and incubated with the respective secondary antibodies and AttoFluor^®^633 phalloidin (dilution 1:100) in 1 % (v/v) goat or donkey serum in PBS w/ Mg^2+^/Ca^2+^ at RT in the dark for 45 min. The samples were rinsed with PBS w/ Mg^2+^/Ca^2+^, mounted on object slides with anti-fading mounting medium, and photo-documented by confocal laser scanning microscopy with a Leica TCS SP5 and either a UV-diode (405 nm), argon laser (488 nm), or helium-neon laser (633 nm) (Leica Microsystems GmbH, Wetzlar, Germany) using Leica LAS. AF image processing software. hCEnC stained for the ECM constituents fibronectin, collagen type IV and laminin is shown in **Supplemental Figures S18**. hCEnC stained for the tight junction protein ZO-1, and the ion-pump Na^+^/K^+^-ATPase α1 are shown in **Supplemental Figures S16 and S17**.

**Histochemistry and immunohistochemistry**

One day after transfer onto de-endothelialized porcine corneas, hCEnC layer were analyzed by histochemical and immunohistochemical staining. Porcine corneas with or without their own corneal endothelium served as controls. Corneas were fixed overnight at 4 °C with 4 % (v/v) formaldehyde in PBS w/ Mg^2+^/Ca^2+^, rinsed two times for 60 min in 0.1 mol l^-1^ Sörensen buffer at RT, and dehydrated in ascending ethanol concentrations to xylene. After embedding in paraffin, 5 µm sections were cut on a rotating microtome (Reichert-Jung 2035, Leica Microsystems) and de-paraffinized by descending ethanol concentrations to distilled water.

**Histochemistry**: Samples were stained with hematoxylin and eosin, dehydrated as described above, mounted in DePex, and photo-documented by light microscopy (OPTIPHOS-2, Nikon, Düsseldorf, Germany).

**Immunohistochemistry**: After blocking with 10 % goat serum in PBS w/ Mg^2+^/Ca^2+^ for 30 min, samples were stained with primary antibodies diluted in PBS w/ Mg^2+^/Ca^2+^ and incubated at 4 °C overnight. Samples were washed, blocked again, and incubated with the secondary antibody as described above. Slides were mounted with an anti-fading mounting medium, and staining was visualized and photo-documented under a BX 60 fluorescence microscope (Olympus Deutschland GmbH, Hamburg, Germany) equipped with a F-View C.C.D. camera run by analySIS imaging software (Soft Imaging System GmbH, Münster, Germany). hCEnC layer was analyzed by histochemical and immunohistochemical staining is shown in **Supplemental Figure S26**.

**Data analysis and graphing**

All the data was processed, and graphs were created using Origin software (version 9.1.0 G, OriginLab Corporation, Northampton, USA). Images were analyzed with ImageJ software (National Institutes of Health (NIH), Maryland U.S.A.). Results are were given as mean ± standard deviation using a paired Student t-test. Statistical significance was defined as p ≤ 0.05.

Supplementary figures:

**
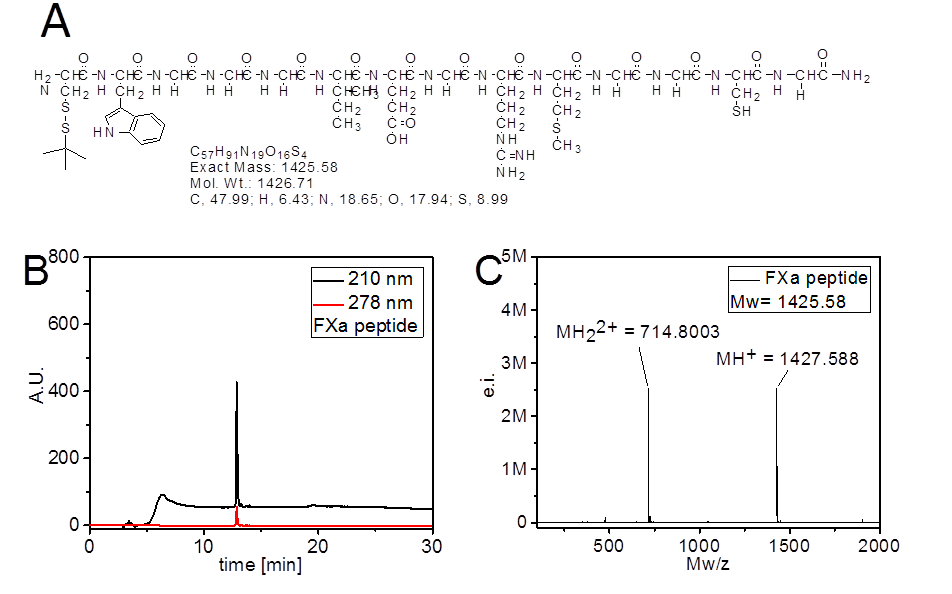
**

**Figure S1.** S**ynthesis of FXa peptide: (A)** Peptide structure of C(StBu)WGGGIEGRMGGCG; **(B)** Analytical HPLC of FXa peptide; **(C)** ESI-MS of purified FXa peptide revealed two species with a molecular weight (Mw) of 714.8 g/mol and 1427.8 g/mol, which corresponds to the double and mono charged FXa peptide ions (calculated Mw = 1425.58 g/mol).

**
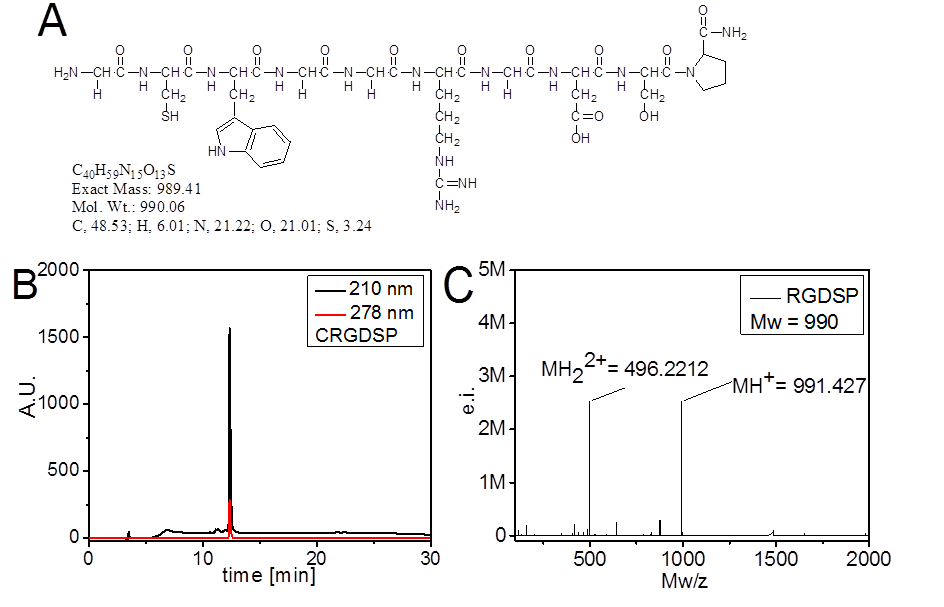
**

**Figure S2. Synthesis of RGD peptide:** **(A)** Peptide structure of GCWGGRGDSP; **(B)** Analytical HPLC of RGD peptide; **(C)** ESI-MS of RGD peptide has revealed two species with a molecular weight of 989.66 g/mol and 493.77 g/mol, which corresponds to mono and double-charged Mw of RGDS peptide (calculated Mw = 989.41 g/mol).

**
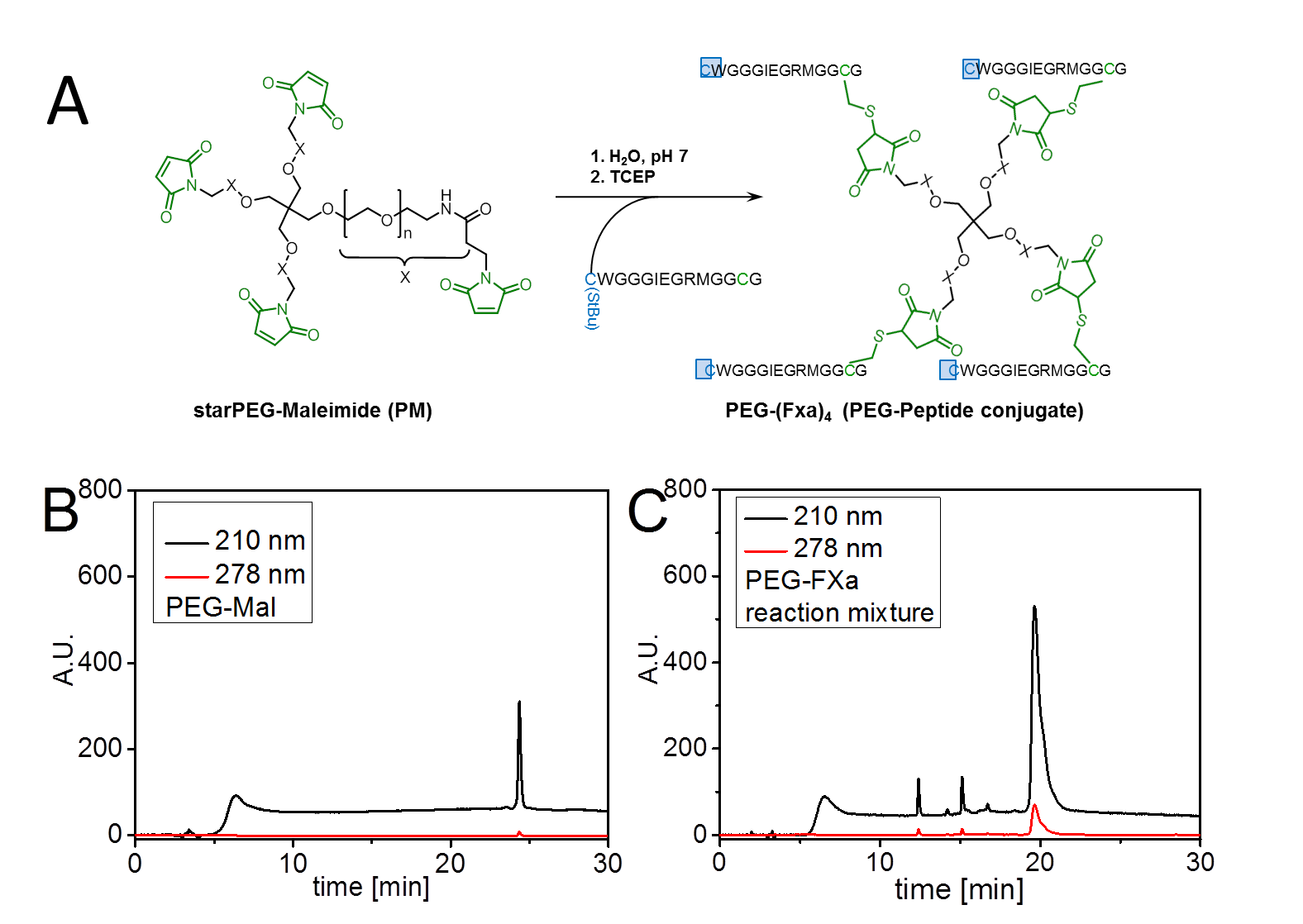
**

**Figure S3.** **Synthesis of PEG-(FXa)_4_ conjugate: (A)** Reaction scheme of the PEG-(FXa)_4_ conjugate formation. **(B)** HPLC analyses of PEG--(maleimide)_4_ ; **(C)** HPLC analyses of a reaction mixture of PEG-(maleimide)_4_ with FXa-peptide.

**
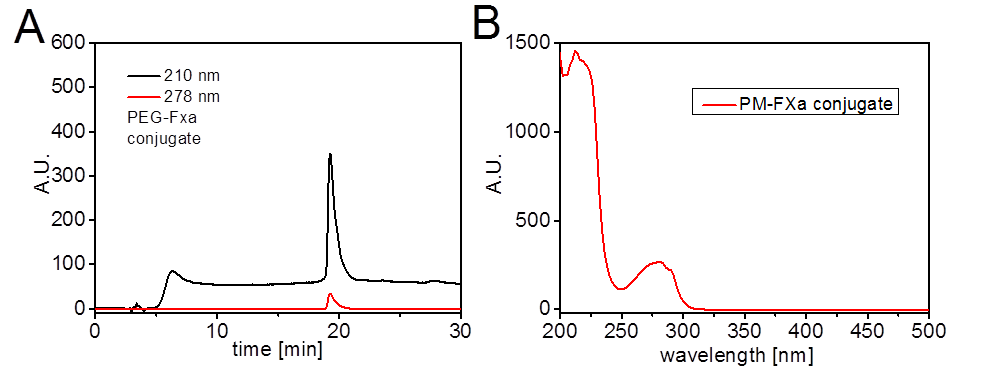
**

**Figure S4.** **Analytical characterization of PEG-(FXa)_4_ conjugate: (A)** HPLC analyses of the purified PEG-(FXa)_4_ conjugate. **(B)** U.V./Vis spectrum of the purified PEG-(FXa)_4_ conjugate.

.

**
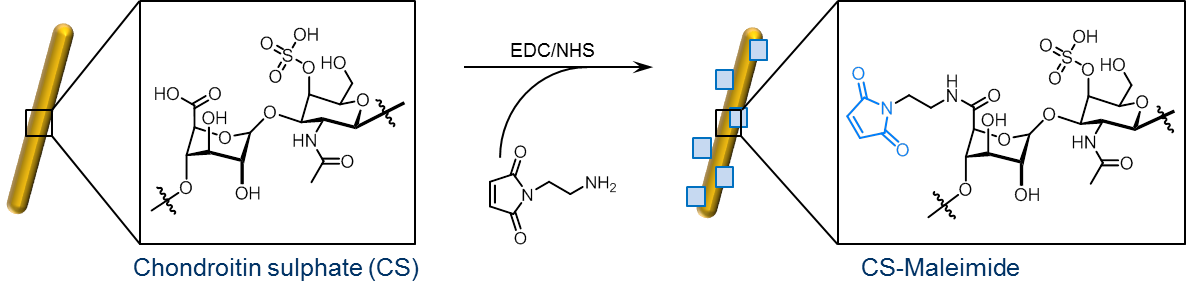
**

**Figure S5.** **Chondroitin sulfate-maleimide conjugate CSMal_6_:** The synthetic scheme the formation of chondroitin sulfate-maleimide conjugate containing six maleimide groups.


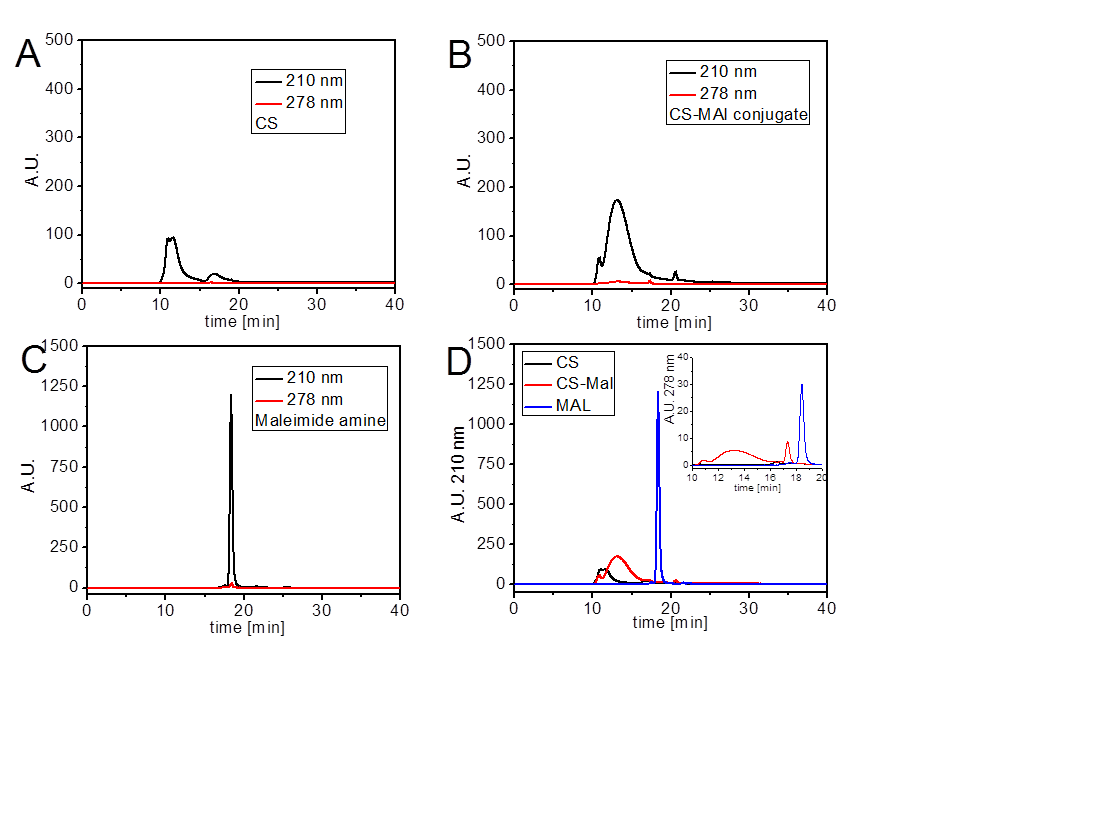


**Figure S6.** **HPSEC characterization of synthesis and purification of chondroitin sulfate-maleimide conjugate CSMal_6_: (A)** Chondrotinsulfate; **(B)** Purified chondroitin sulfate-maleimide conjugate CSMal_6_ with 6 maleimide groups per chondroitin sulfate molecule; **(C)** Maleimide-amine; **(D)** Overlap of the HPSEC chromatograms of the chondroitin sulfate, maleimide-amine and their conjugate revealed the purity of the product (no unreacted maleimide present in the CSMal_6_ sample) and the completion of the reaction. The absorption of the CSMal_6_conjugate has characteristic absorbance of maleimides at 278 nm (insertion) while chondroitin sulfate does not (concentration 2 mg/ml, injection volume: 50 µl).

.

**
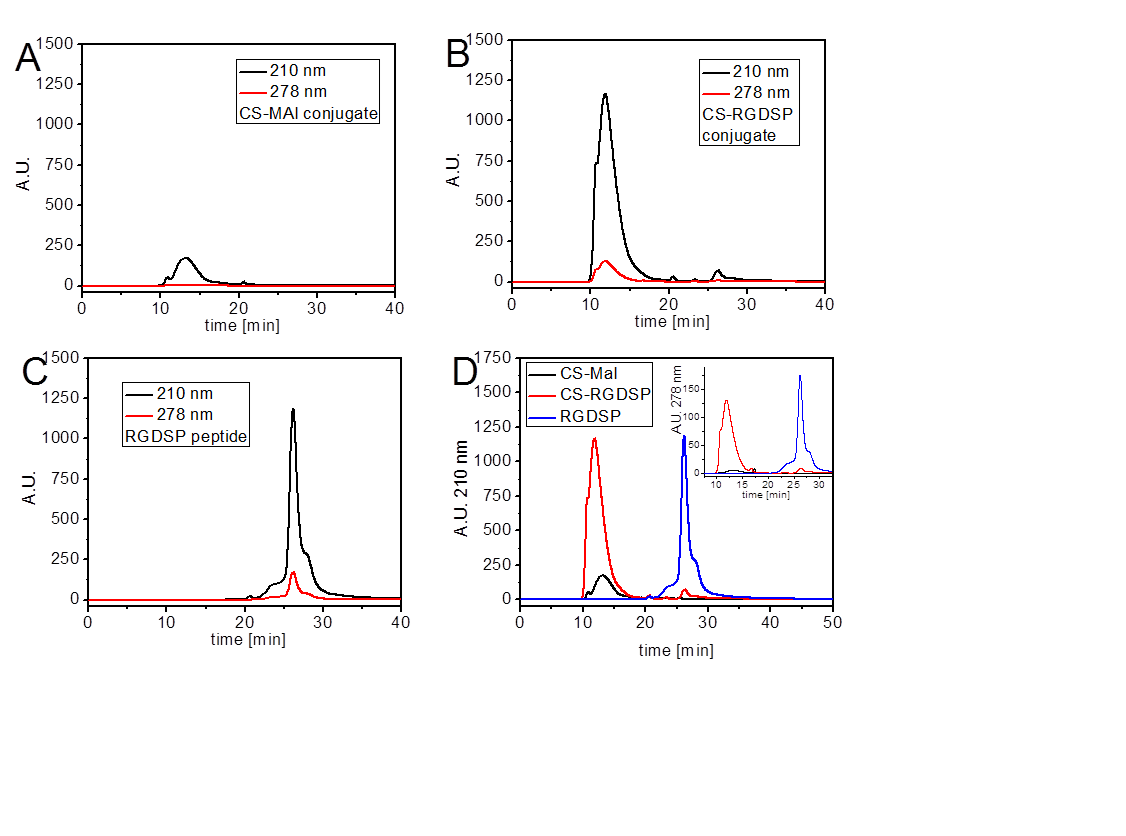
**

**Figure S7.** **HPSEC evaluation of the reactivity of maleimide groups within chondroitin sulfate-maleimide conjugate CSMal_6_: (A)** Purified chondroitin sulfate-maleimide conjugate CSMal_6_; **(B)** Reaction mixture of CSMal_6_ conjugate with an equimolar amount of RGD peptide; **(C)** apo-peptide RGD; **(D)** Overlap of the HPSEC chromatograms of the CSMal_6_ conjugate, apo-peptide RGD, and their reaction mixture revealed the completion of the reaction. The formed CS (RGD)_6_ product should show one main peak at 13.5 min, which is characteristic of CSMal6 but with much stronger absorption and a very small peak at 27 min, corresponding to the unreacted RGD peptide. The total absorption of the heparin-peptide conjugate at 278 nm (insertion), when compared to the absorption of unreacted peptide RGDS, revealed more than 95% reactivity of the CSMal_6_ (concentration: 2 mg/ml, injection volume: 50 µl).


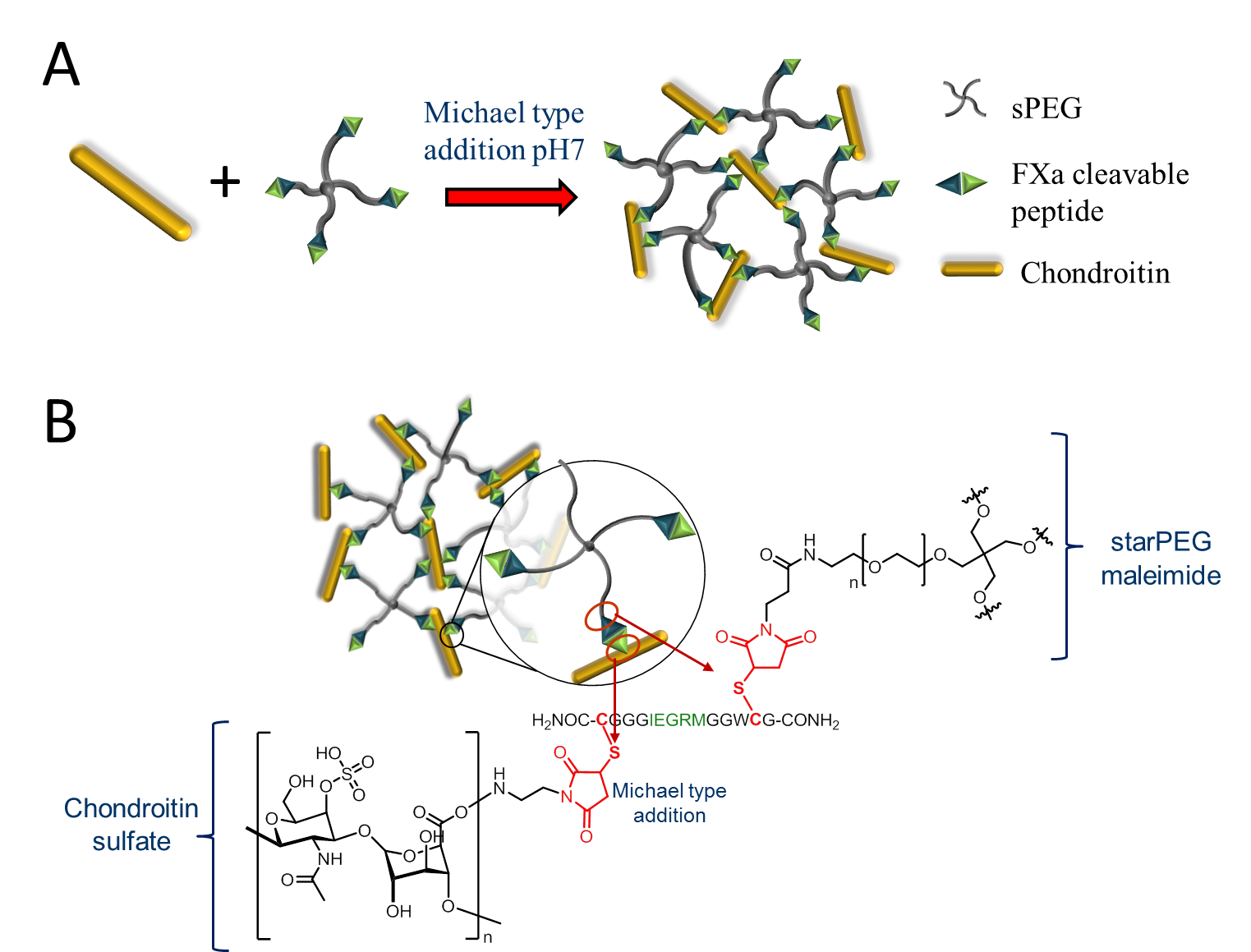


**Figure S8. The schematic view of hydrogel network formation:** Hydrogel network is formed by Michael type addition via the formation of peptide crosslinking points.


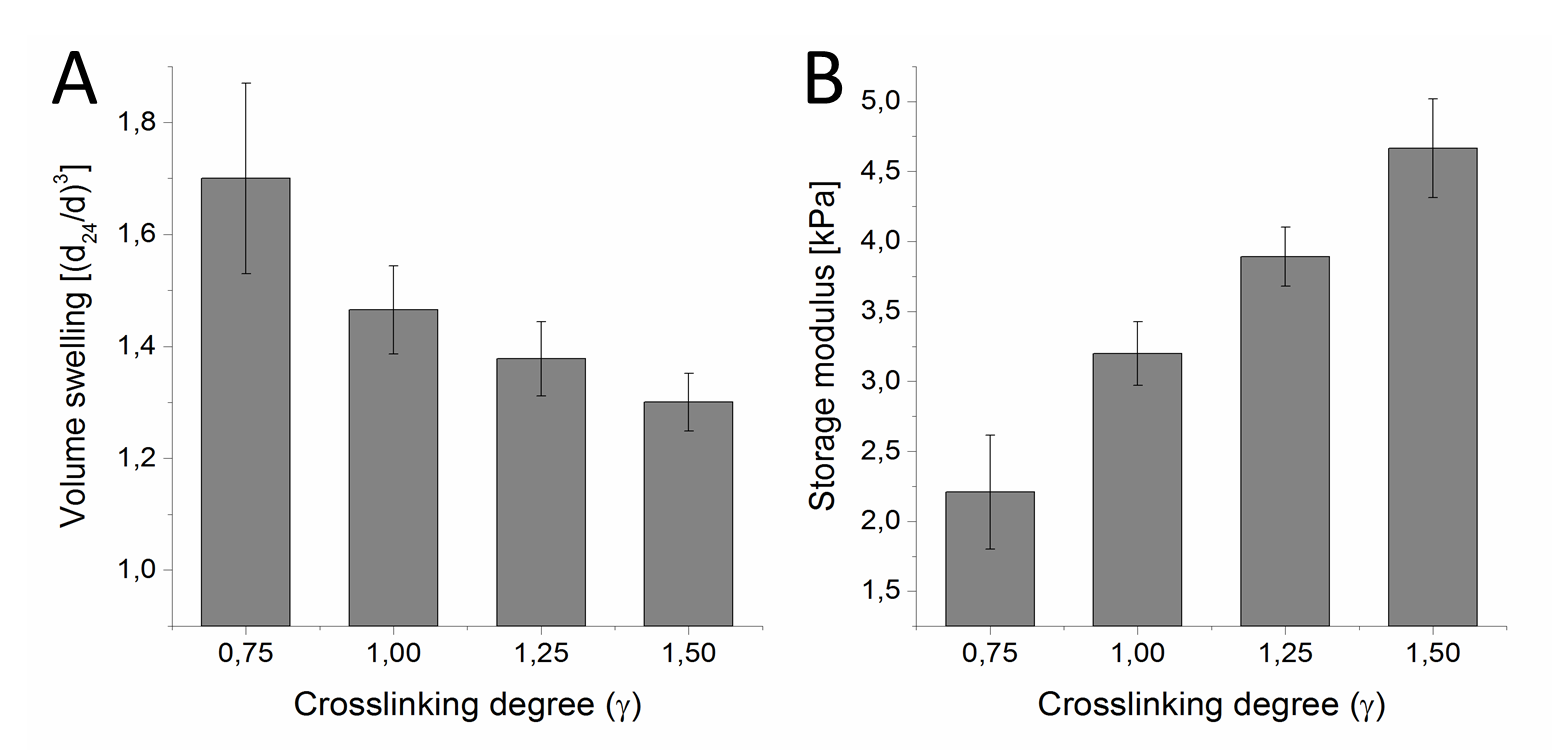


**Figure S9. Mechanical properties of PEG/ CSMal_6_** **hydrogels as a function of crosslinking degree:** **(A)** the influence of the crosslinking degree (molar ratio of the building blocks PEG/ CSMal_6_ = γ) on swelling degree; **(B**) the influence of the crosslinking degree (γ) on the stiffness.

**
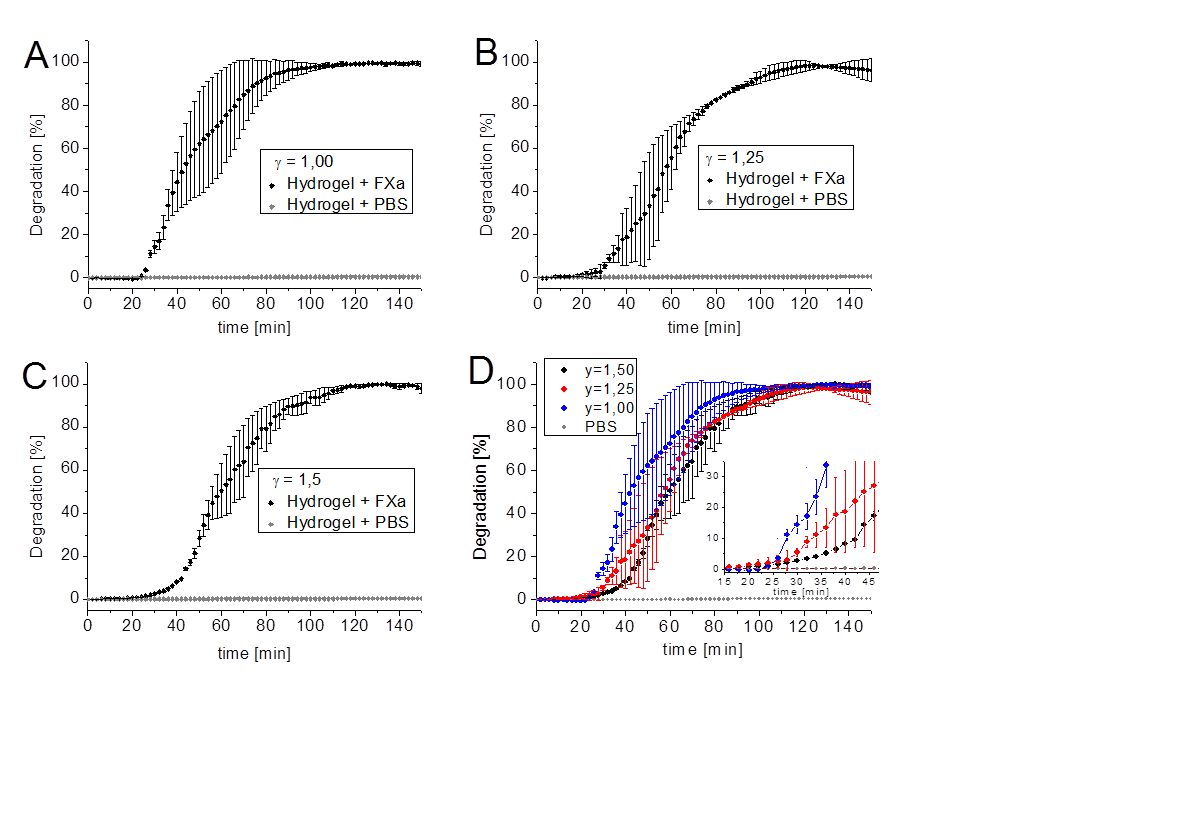
**

**Figure S10. Enzymatic hydrogel degradation:** Fast enzymatic decomposition of FXa-cleavable hydrogels of crosslinking degree **(A)** γ = 1; **(B**) γ = 1.25; **(C)** γ = 1.5 monitored by UV/Vis spectroscopy of released peptide in 900 nM FXa solution in PBS (all gels remain stable in PBS which is used as a negative control); **(D**) Overlap of the degradation curves of FXa-cleavable hydrogels revealed the dependence of the degradation rate and hydrogel crosslinking degree. Higher crosslinking degree results in a slower degradation rate (insertion).

**
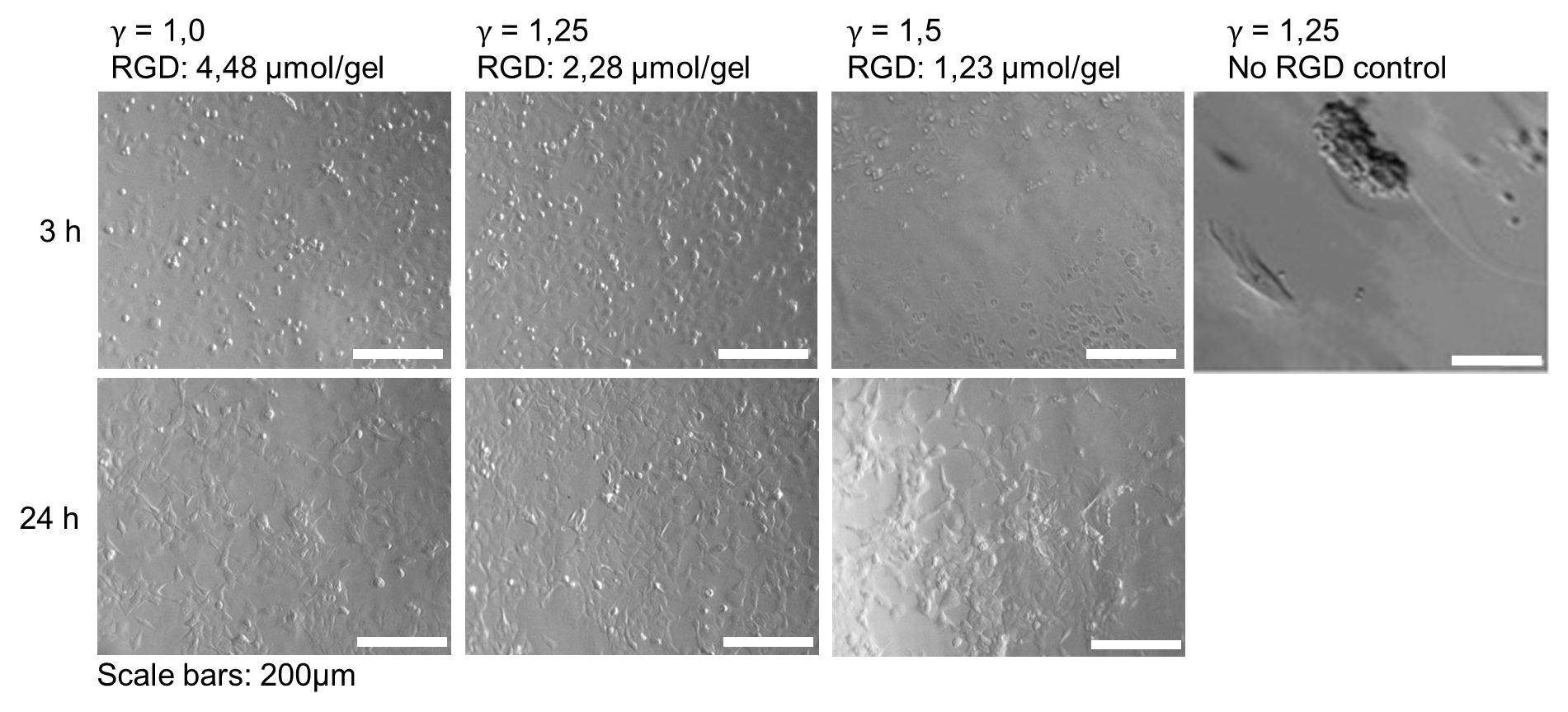
**

### Figure S11. Human corneal endothelial cell culture on FXa-degradable hydrogels: Light microscopy images of hCEnC growing on FXa cleavable PEG-chondroitin hydrogel. Adhesive RGD peptide was essential for cell spreading. The negative control with the lack of RGD peptide has revealed poor cell adhering and no spreading.

**
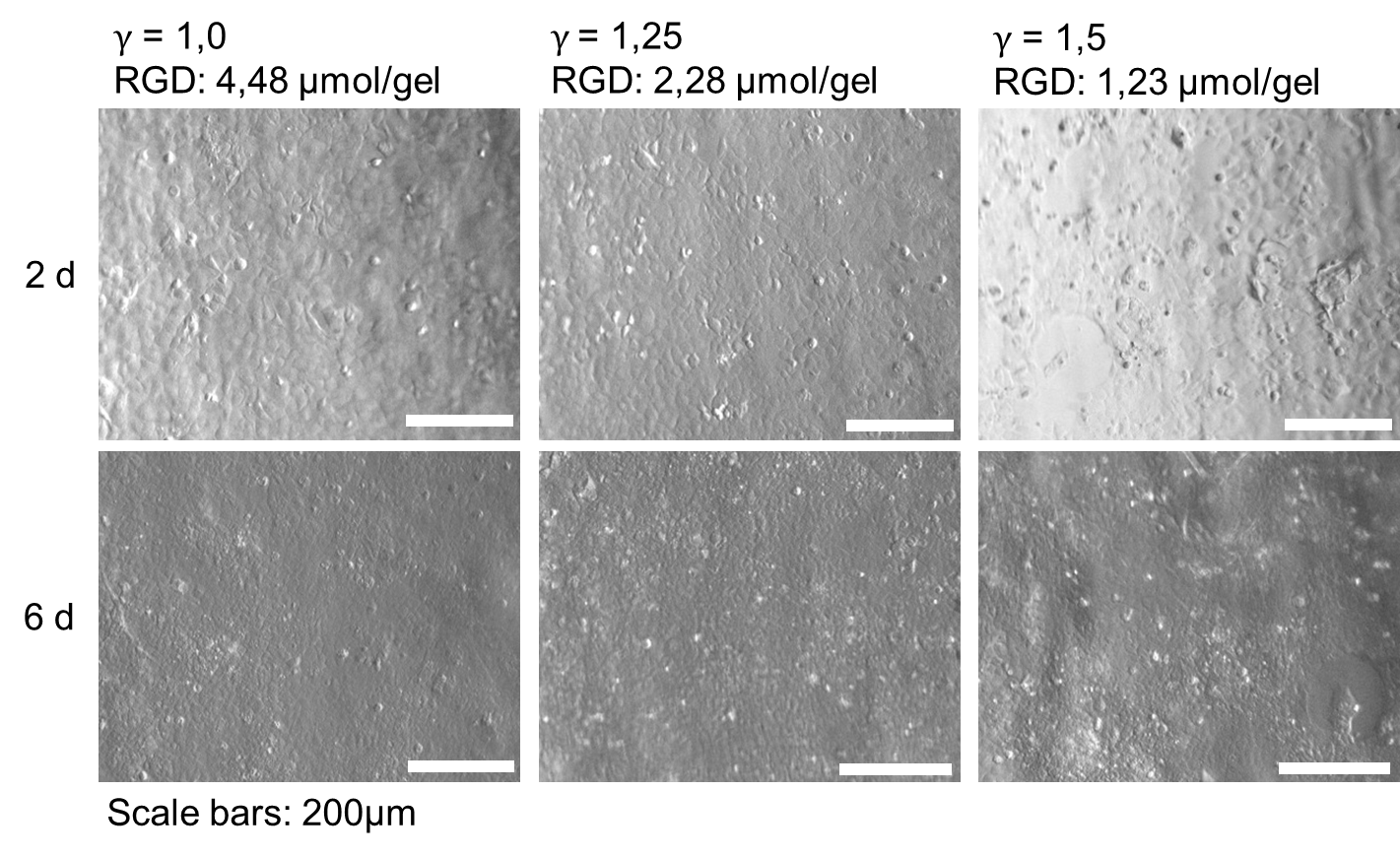
**

### Figure S12. Long-term hCEnC culture on FXa-degradable hydrogels: FXa-cleavable hydrogels of crosslinking degree γ = 1.25 have been found the most suitable for hCEnC culturing as a nearly confluent cell layer was formed after 48 hours.

**
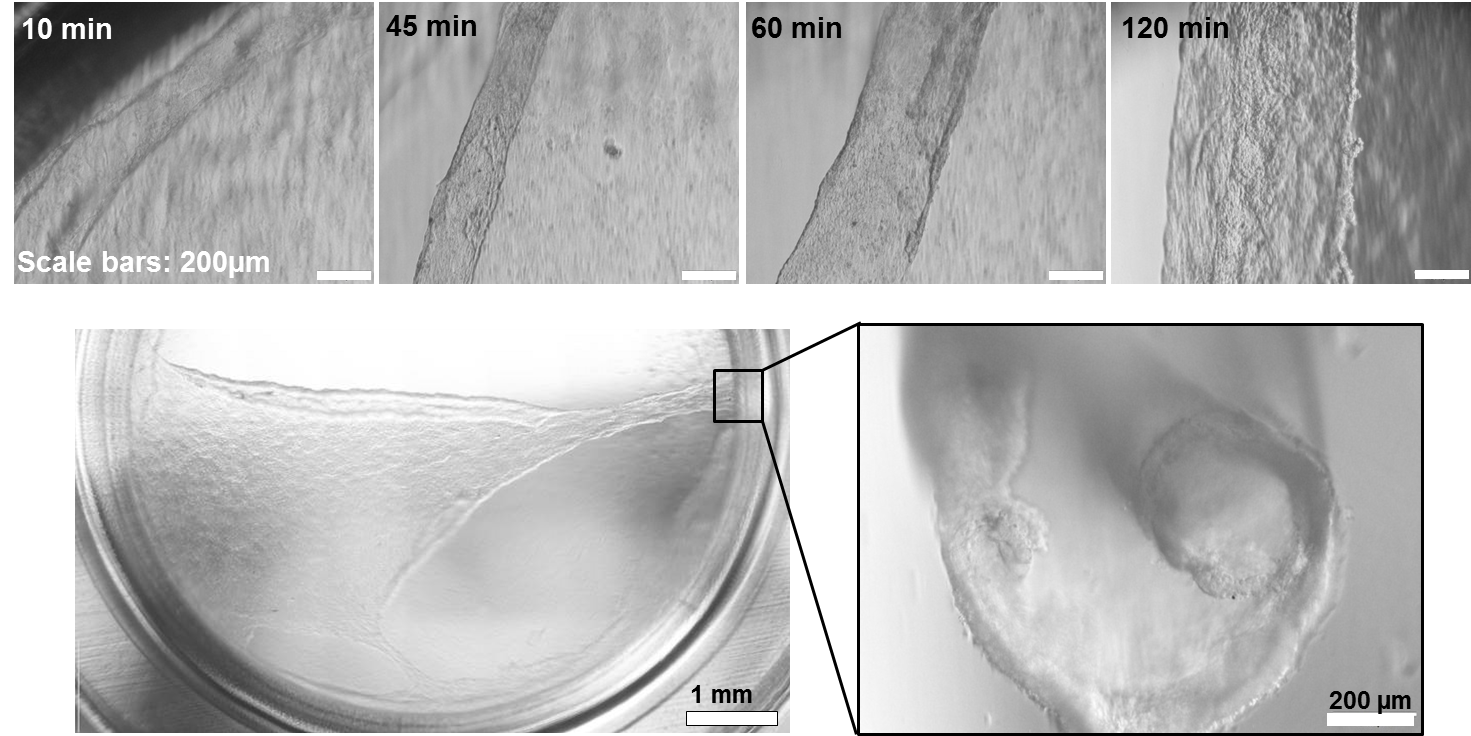
**

**Figure S13. hCEnC layers formation:** Light microscopy images of the hCEnC layers formation via the hydrogel degradation (Human Endothelial-SFM w/ 450 nM Factor-Xa Endoprotease, at 37 °C), complete degradation occurred in 120 min.


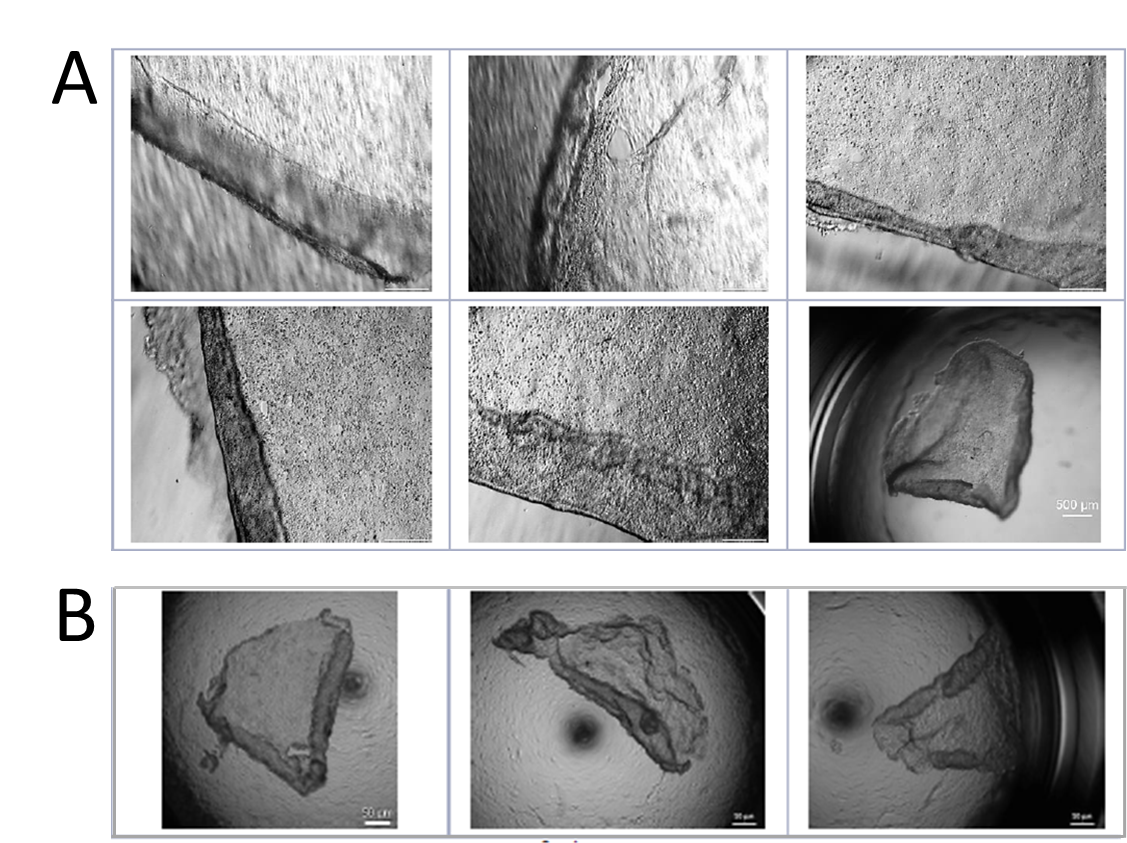


**Figure S14. hCEnC layers formation:** **(A)** Light microscopy images of the hCEnC layers formation via the hydrogel degradation (Human Endothelial-SFM w/ 900 nM Factor-Xa Endoprotease, at 37 °C), complete degradation occurred in 45 min**; (B)** Light microscopy images various examples of the formed hCEnC layer.

**
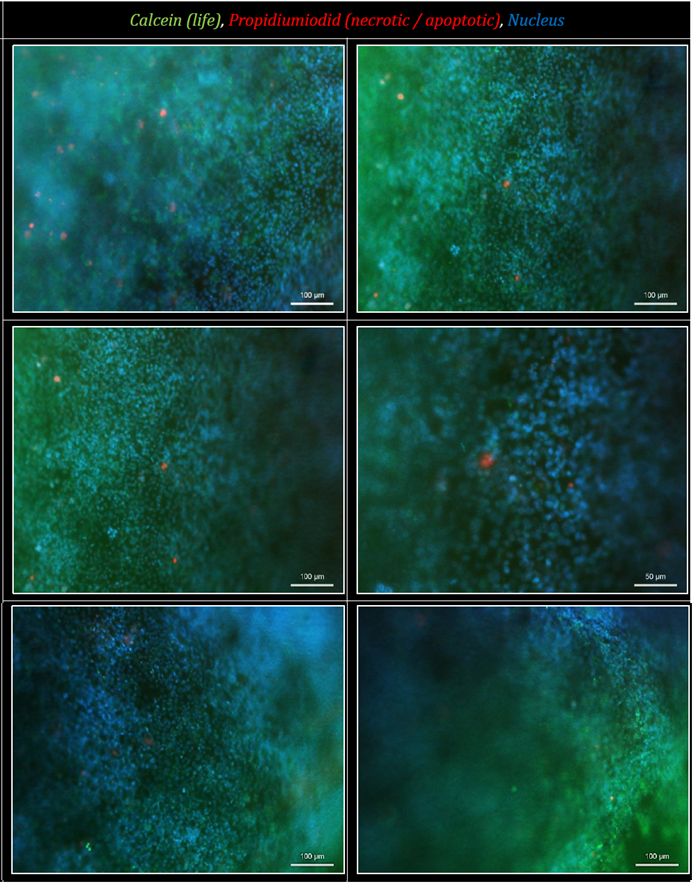
**

**Figure S15. Viability (live/dead) staining** **human corneal endothelial cell culture:** hCEnC culture show nearly >99% survival after 6 days culturing on RGD-containing, FXa-cleavable hydrogels. The survival rate did not dependent on the crosslinking degree. Green = viable cells; red = necrotic cells, blue = cell nuclei.

. **
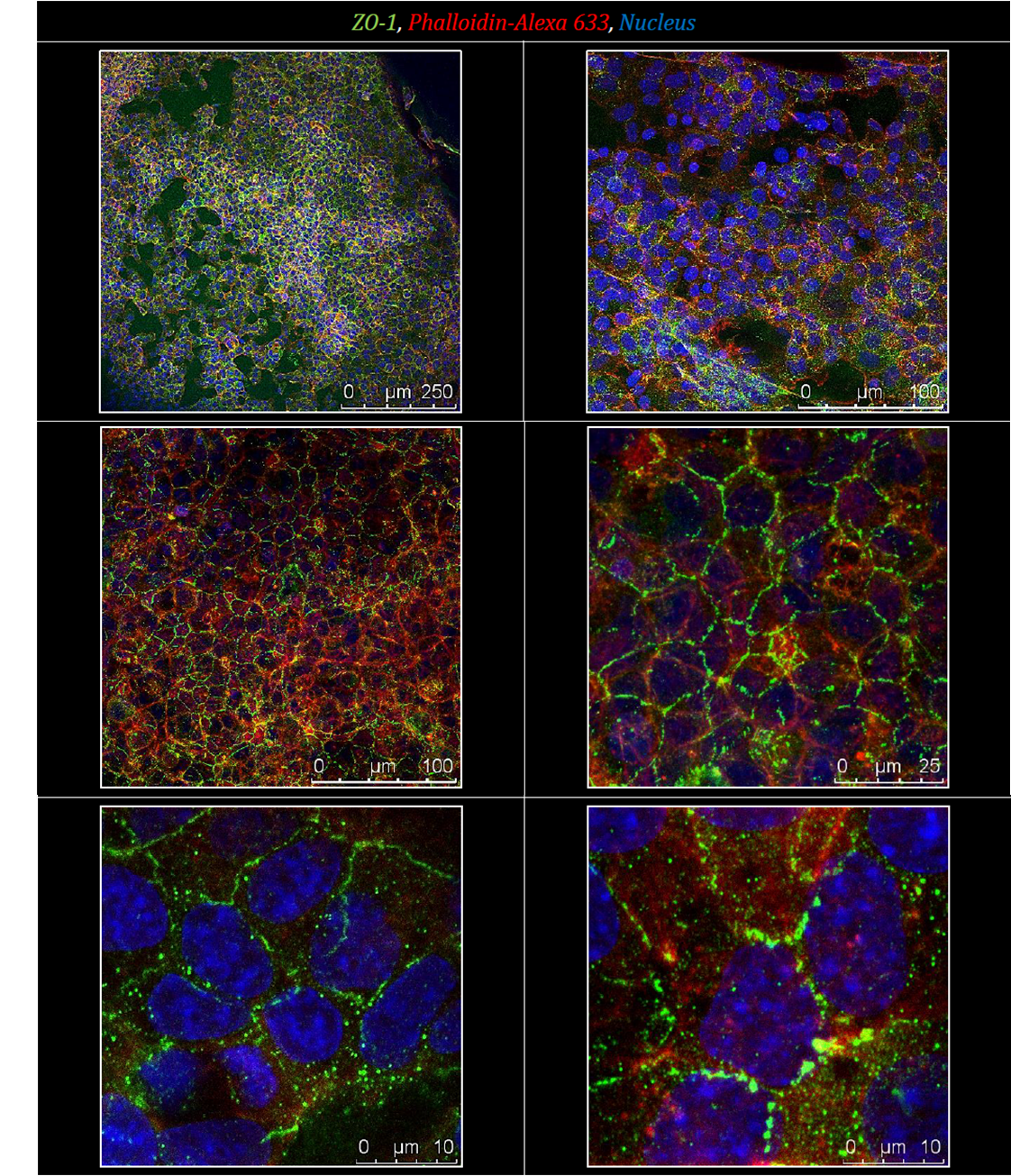
**

**Figure S16. hCEnC culture on FXa-cleavable hydrogel stained for the tight junction protein ZO-1:** hCEnC cultured for seven days on FXa-degradable hydrogels were positive for tight junction Zonula occludens-1 (ZO-1), which were localized at the lateral cell membranes. Antigens of interest are shown in green (Alexa Fluor^®^488), F-actin fibers in red (Phalloidin), and the nuclei in blue (Hoechst 33342).

**
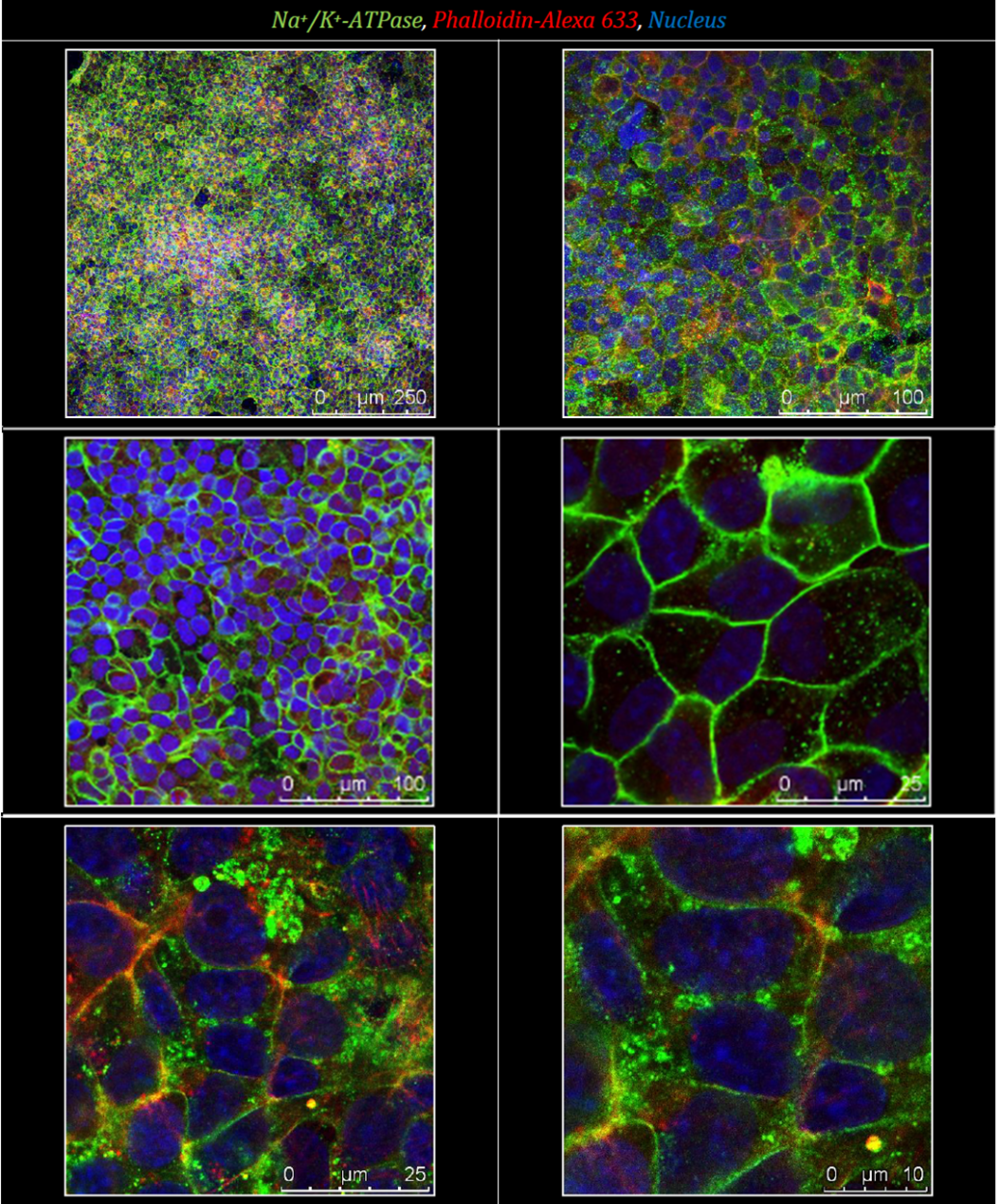
**

**Figure S17. hCEnC culture on FXa-cleavable hydrogel stained for the ion-pump Na^+^/K^+^-ATPase α1:** hCEnC cultured for seven days on FXa-degradable hydrogels were positive for the ion-pump Na^+^/K^+^-ATPase α1 which were localized at the lateral cell membranes. Antigens of interest are shown in green (Alexa Fluor^®^488), F-actin fibers in red (Phalloidin), and the nuclei in blue (Hoechst 33342).

**
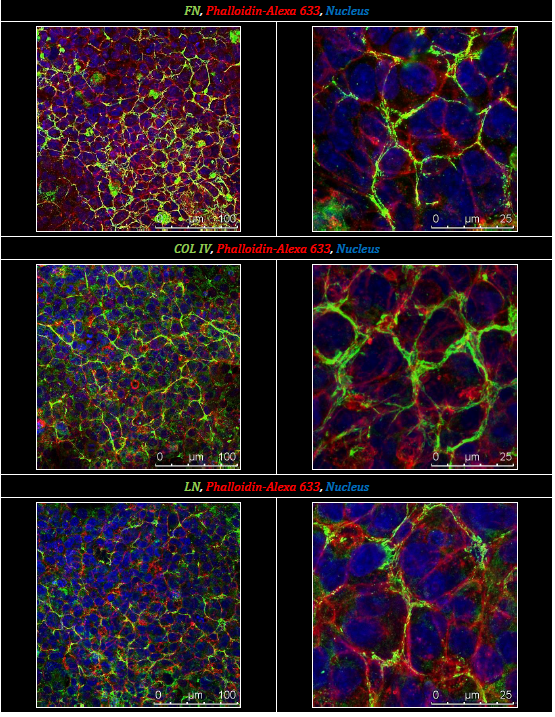
**

**Figure S18: hCEnC culture on FXa-cleavable hydrogel stained for the ECM constituents fibronectin, collagen type IV, and laminin:** hCEnC cultured for seven days on FXa-degradable hydrogels expressed fine fibers of laminin, collagen type IV, and fibronectin as typical components of the extracellular matrix of the corneal endothelium. Antigens of interest are shown in green (Alexa Fluor^®^488), F-actin fibers in red (Phalloidin), and the nuclei in blue (Hoechst 33342).

**
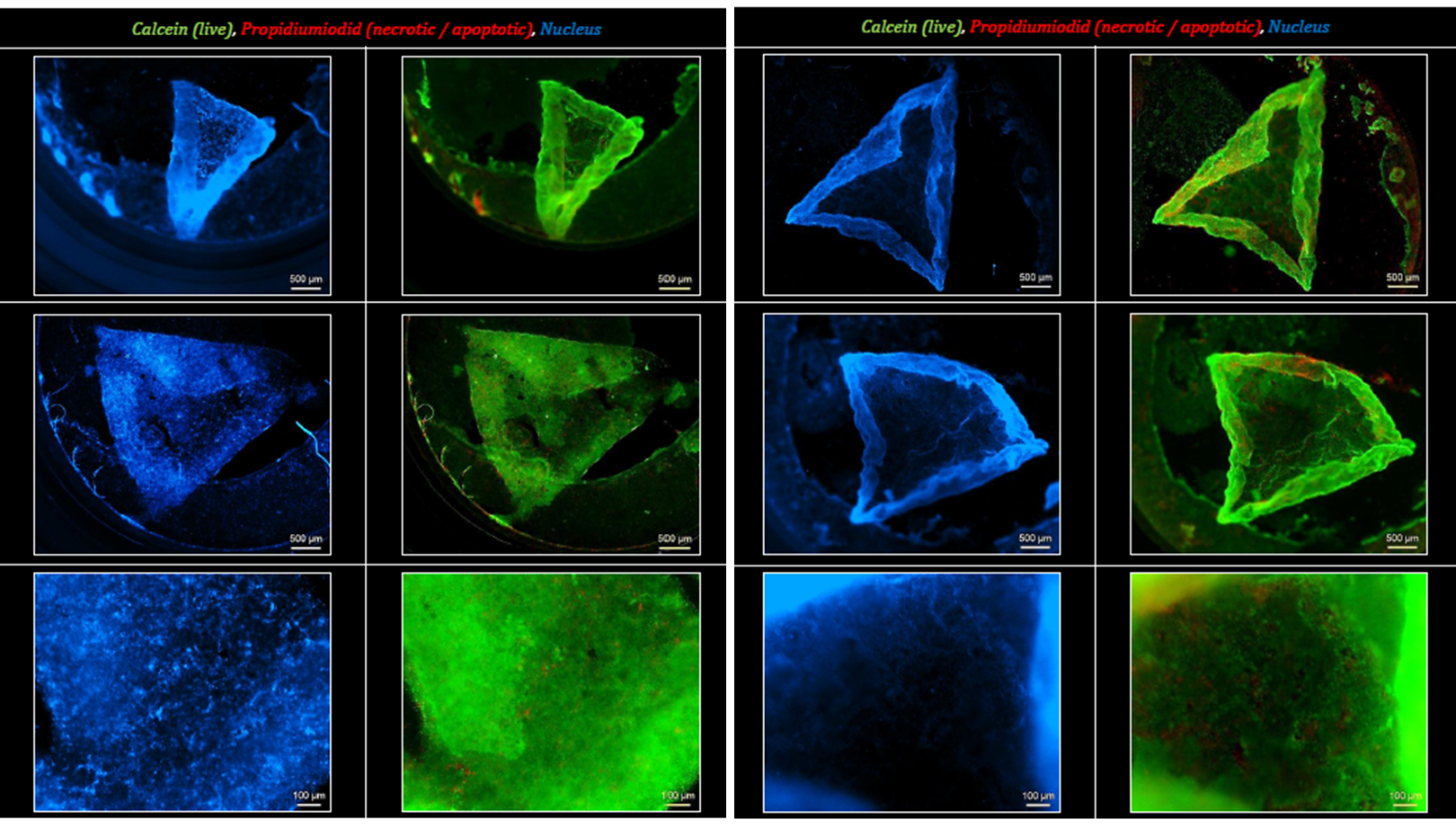
**

**Figure S19. Viability (live/dead) staining** **of formed hCEnC tissue layers:** Life-dead-staining of the released cell layer showed that the majority of the cells were still viable after the detachment. Viable cells are shown in green (Calcein-AM) and necrotic cells in red (PI).

.

**
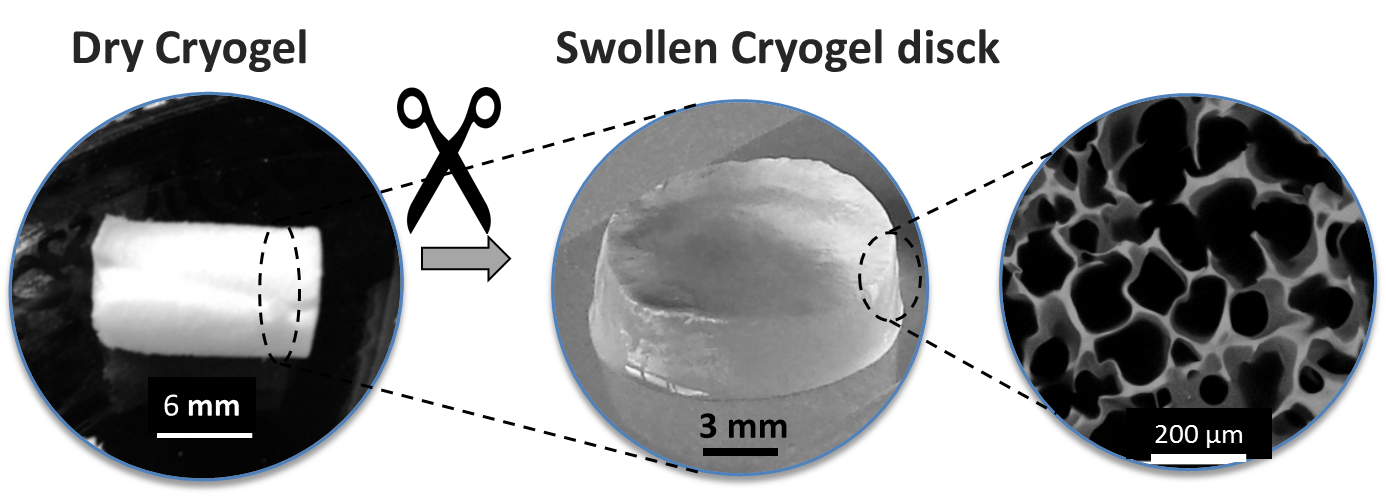
**

**Figure S20. The schematic view of macroporous hydrogel (Cryogel) disc formation for tissue transfer:** The hydrogel discs were formed by 1 mm cuts of the 6 mm in diameter cryogel cylinders. The average pore size distribution was below 50 microns.

**
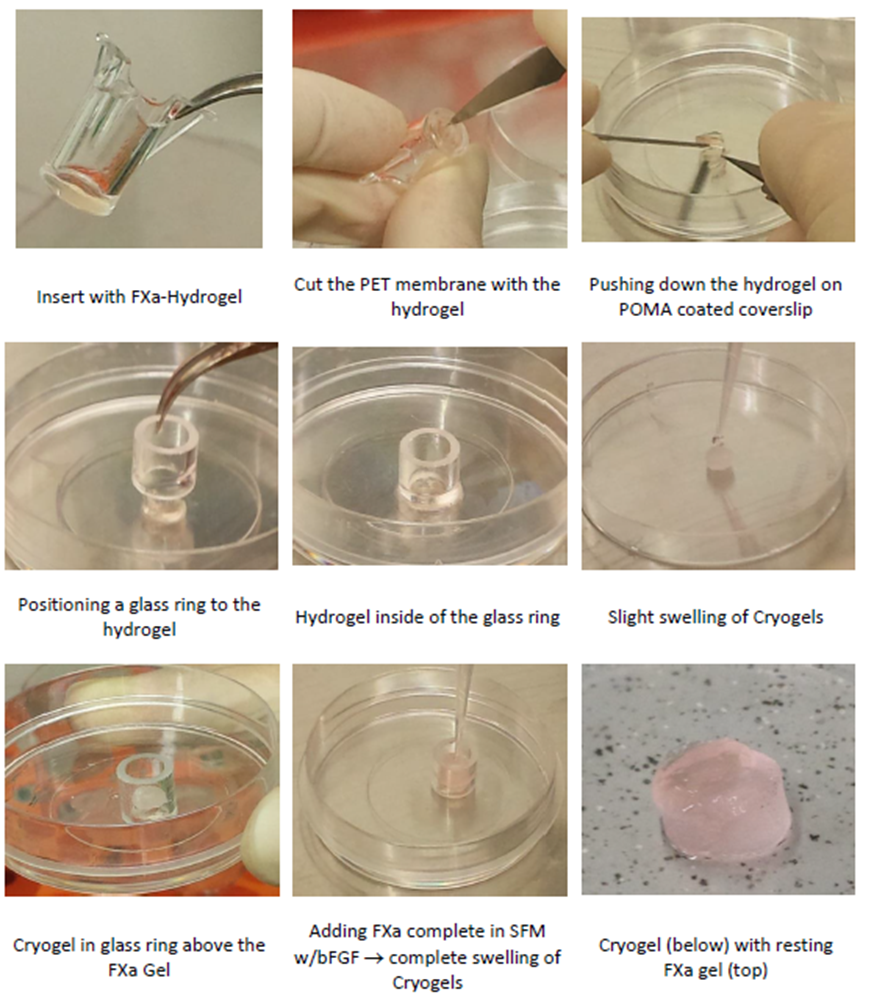
**

**Figure S21. Preparation of macroporous hydrogel (cryogel) as a transfer tool.** Macroporous biohybrid hydrogels composed of starPEG and heparin with a cross-linking degree of γ = 2 were prepared with the adjusted protocol described by Welzel *et al*. [5] in order to fit the glass ring.

**
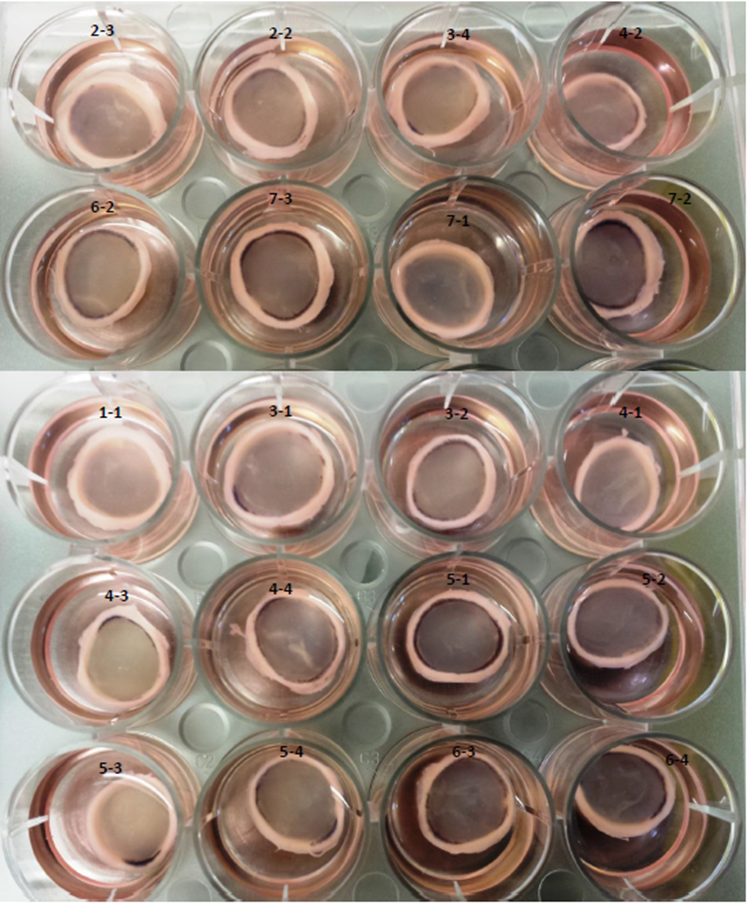
**

**Figure S22. hCEnC layers transplanted onto de-endothelialized porcine corneas.** Released cellular monolayers were stabilized by discs of macroporous biohybrid hydrogels and transferred onto the new concave target surface – a de-endothelialized porcine cornea.

**
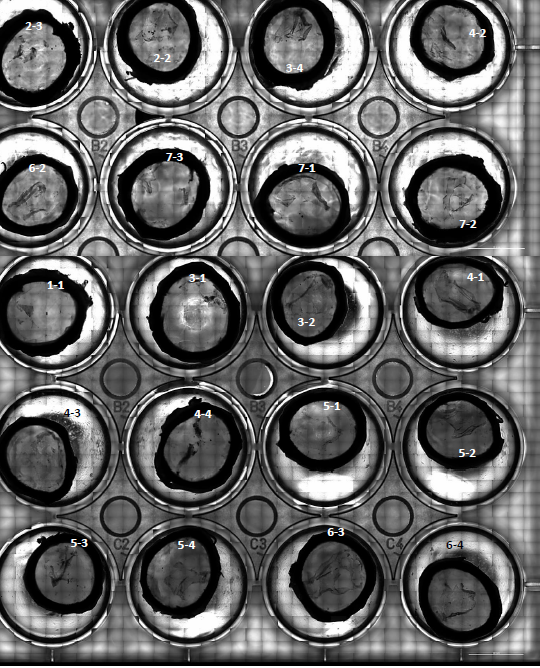
**

**Figure S23. hCEnC layers transplanted onto de-endothelialized porcine corneas.** High resolution stitched overview light microscopy images. Released cellular monolayers were stabilized by discs of macroporous biohybrid hydrogels and transferred onto the new concave target surface – a de-endothelialized porcine cornea.

**
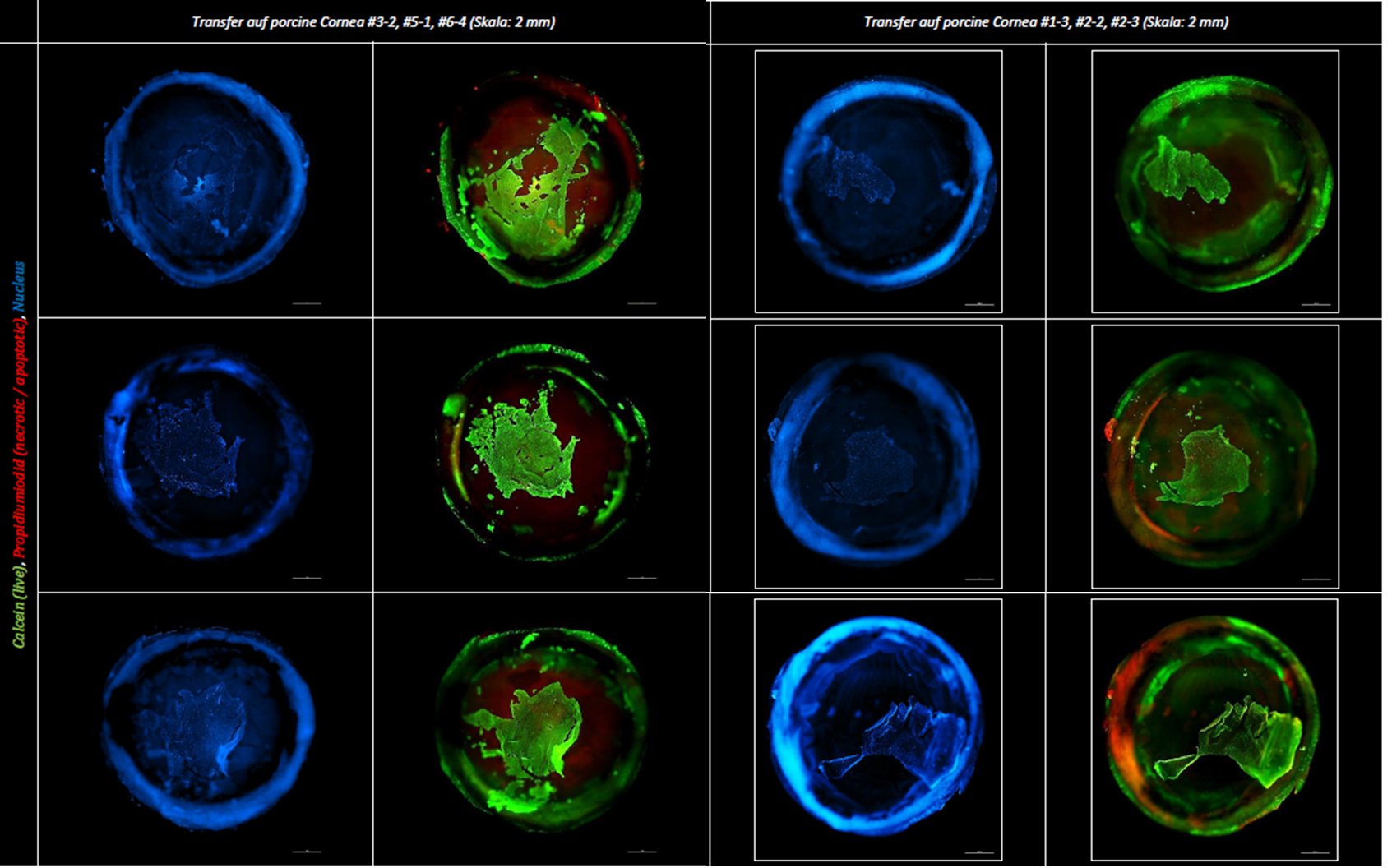
**

**Figure S24.** **Viability staining porcine corneas with implanted hCEnC tissue layer:** Different porcine corneas after the transfer (24 h) of the engineered hCEnC layer. Green = viable cells; red = necrotic cells, blue = cell nuclei


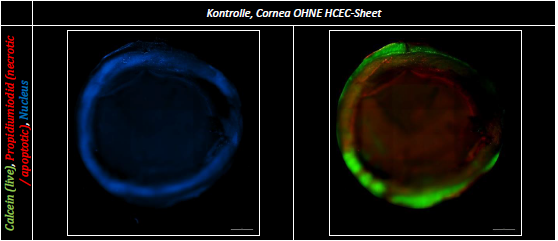


**Figure S25. Porcine corneas without corneal endothelium after viability staining:** Porcine cornea without corneal endothelium after de-endothelialization. Green = viable cells; red = necrotic cells, blue = cell nuclei, scale bar?


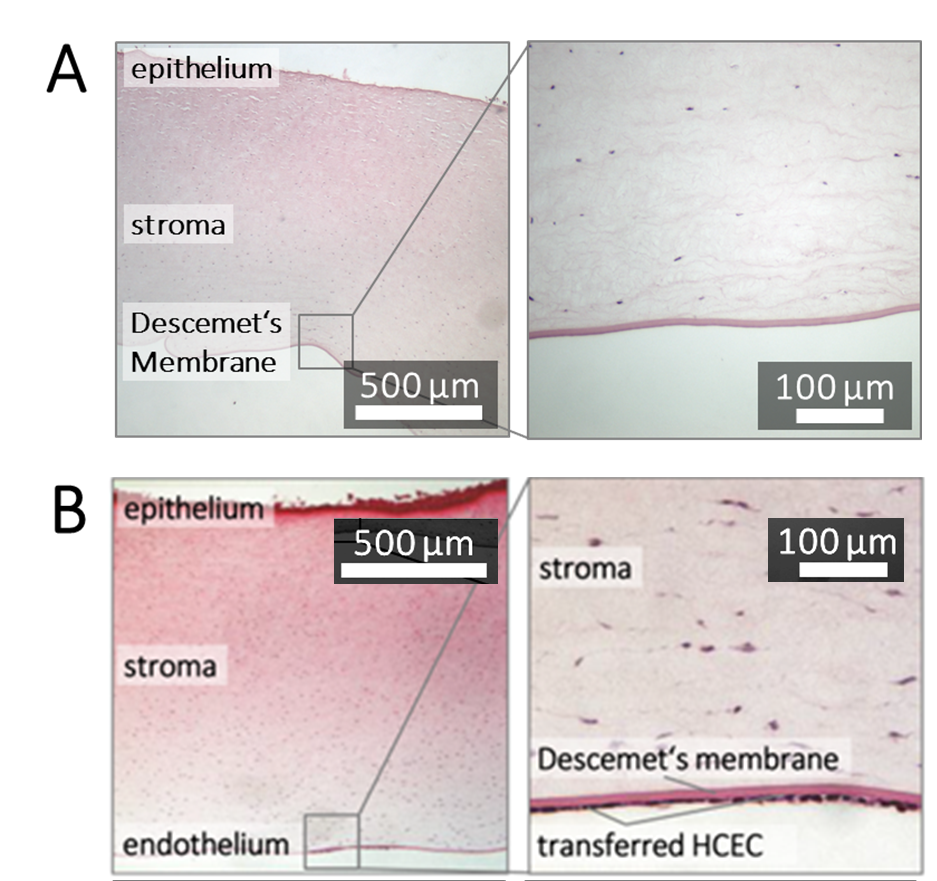


**Figure S26. Histochemistry and immunohistochemistry of hCEnC layers transplanted onto de-endothelialized porcine corneas: (A)** Porcine corneas without corneal endothelium after viability staining; **(B)** One day after transfer onto de-endothelialized porcine corneas, hCEnC layer were analyzed by histochemical and immunohistochemical staining. Porcine corneas with or without their own corneal endothelium served as controls**.**

**
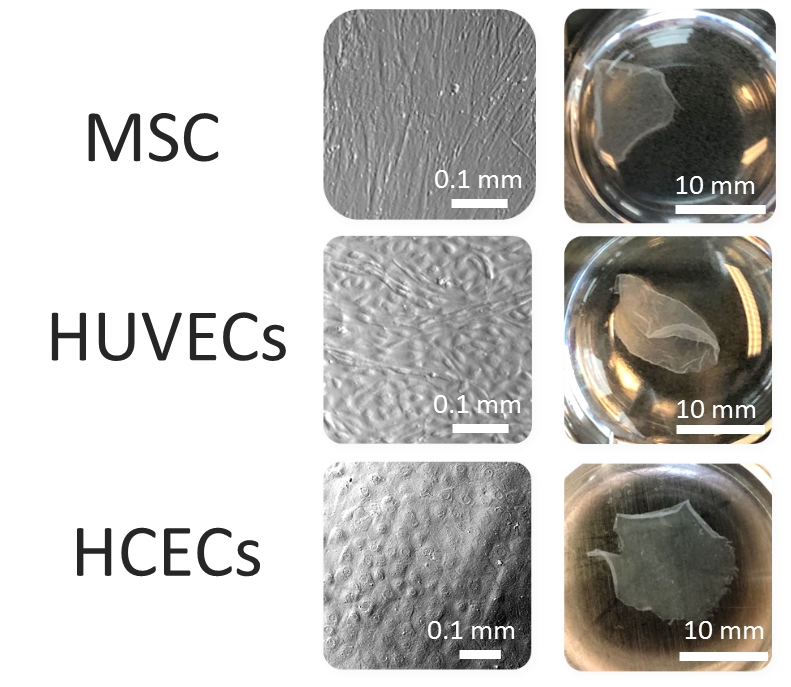
**

**Figure S27. Examples of cell sheet formation and harvesting:** primary mesenchymal stem cells (MSC), primary human umbilical vein endothelial cells (HUVEC), and human corneal endothelial cells (hCEnC) utilizing FXa-degradable biohybrid hydrogel substrates. Presented are phase contrast (left), macroscopic (right),

**Table S1 Main proteases (TAG Enzymes) used to remove fusion tags.**

| **proteases (commonly used abbreviation)** | **The most often-used sequence** |
| --- | --- |
| Tobacco etch virus protease (TEV) | ENLYFQ/ |
| Human rhinovirus 3C protein (3C) | ETLFQ/ EVLFQ/ TLFQ/GP |
| Factor Xa (Xa) | IEGR/ |
| Thr Thrombin | LVPRGS/ FXRS/ |
| EntK Enterokinase | DDDDK/ |
| Caspase Caspase-3 | DXXD/ |
| PreScission | LEVLFQ/ |
| Sortase A | LPETG |

**X means any residues of proline, Pipecolic acid, Cyclohexylalanine or Cyclohexylglycin.*

**References and Notes:**

**References:**

1. Tsurkan M. V. et al. Defined polymer–peptide conjugates to form cell-instructive starPEG–heparin matrices in situ. *Adv. Mater.* **25**, 2606–2610 (2013).

2. Tsurkan M. V., et al. Chemoselective peptide functionalization of starPEG-GAG hydrogels *Bioconjugate Chemistry* **25**, 1942-195 (2014).

3. Tsurkan M. V. et al. Photopatterning of multifunctional hydrogels to direct adult neural precursor cells. *Adv. Healthc. Mater.* **4**, 516–521 (2015).

4. Pompe T. et al. Immobilization of growth factors on solid supports for the modulation of stem cell fate. *Nat Protoc* **5**, 1042-1050 (2010).

5. Welzel P. B. et al. Macroporous starPEG-heparin cryogels. *Biomacromolecules* **13**, 2349−2358 (2012).

6. Valtink M. et al. Two clonal cell lines of immortalized human corneal endothelial cells show either differentiated or precursor cell characteristics. *Cells. Tissues. Organs* **187**, 286–94 (2008).

7. Teichmann J. et al. Thermo-responsive cell culture carriers based on poly (vinyl methyl ether) - the effect of biomolecular ligands to balance cell adhesion and stimulated detachment. *Sci. Technol. Adv. Mater.* **16**, 45003 (13pp) (2015).

**Author Contributions**

MVT initiated and designed the study, performed the hydrogel synthesis and the related analysis, supervised RS, and wrote the manuscript. JB designed and performed the cornea transfer experiments, supervised RS and DP, and wrote the manuscript. RS performed the hydrogel synthesis and the related analysis, cell experiments, and analyzed the data. DP and SDT helped with cell and cornea transfer experiments. PBW designed and synthesized the cyrogel tool, discussed data, edited the manuscript. MFM, CW discussed data, and edited the manuscript.
